# RhCMV expands CCR5^+^ memory T cells and promotes SIV reservoir seeding in the gut mucosa

**DOI:** 10.1172/jci.insight.198743

**Published:** 2025-11-25

**Authors:** Chrysostomos Perdios, Naveen Suresh Babu, Celeste D. Coleman, Anna T. Brown, Shevon N. Alexander, Matilda J. Moström, Carolina Allers, Lara Doyle-Meyers, Christine M. Fennessey, Lori A. Rowe, Brandon F. Keele, Amitinder Kaur, Michael L. Freeman, Joseph C. Mudd

**Affiliations:** 1 Division of Immunology and; 2 Division of Veterinary Medicine, Tulane National Primate Research Center, Covington, Louisiana, USA.; 3 AIDS and Cancer Virus Program, Frederick National Laboratory for Cancer Research, Frederick, Maryland, USA.; 4 Division of Microbiology, Tulane National Primate Research Center, Covington, Louisiana, USA.; 5 Rustbelt Center for AIDS Research, Division of Infectious Diseases & HIV Medicine, Department of Medicine, Case Western Reserve University, Cleveland, Ohio, USA.

**Keywords:** AIDS/HIV, Immunology, Adaptive immunity, T cells, Th1 response

## Abstract

Cytomegalovirus (CMV) is a prevalent β-herpesvirus that persists asymptomatically in immunocompetent hosts. In people with HIV-1 (PWH), CMV is associated with HIV-1 persistence and particular inflammatory-related comorbidities. The true causative role of CMV in HIV-associated pathologies, however, remains unclear given that nearly all PWH are coinfected with CMV. In this study, we examined acute phase immune and virological dynamics in cohorts of SIV-infected rhesus macaques (RMs) that were naturally seropositive or -negative for rhesus CMV (RhCMV). We observed prior to SIV, RhCMV expanded a polyclonal population of target CCR5^+^CD4^+^ T cells in gut and lymph nodes that expressed the chemotactic receptor CXCR3 and were largely not specific for RhCMV. Upon SIV infection, RhCMV^+^ RMs exhibited higher peak viremia and elevated levels of SIV DNA in the upper and lower intestine. Greater seeding of SIV DNA was associated with a maintenance of CCR5-expressing CD4^+^ T cells that were enriched within the RhCMV^+^ gut along a CXCR3/CXCL9 chemotactic axis. Overall, the data suggest that RhCMV can promote SIV susceptibility within a diverse, polyclonal pool of CD4^+^ T cells that are not entirely RhCMV specific.

## Abstract 2

Rhesus cytomegalovirus expands CCR5+CXCR3+CD4+ T cells, enhancing SIV replication and seeding in mucosal tissues during acute infection.

## Introduction

Cytomegalovirus (CMV; HHV-5), a β-herpesvirus, commonly infects humans from a young age. The global anti-CMV IgG seroprevalence is high with a worldwide average of 83%, although this can vary significantly between countries (1). There is also a high geographic co-prevalence of CMV infection with HIV-1, particularly in sub-Saharan African countries (2). Only within immunocompromised settings does CMV manifest overt disease (3). Nonetheless, maintaining CMV in an asymptomatic state presents a significant burden to the immune system. CMV-specific CD4^+^ and CD8^+^ T cells dominate the immune landscape in blood, as approximately 10% of circulating T cells can be reactive to CMV peptides in seropositive healthy adults (4). These cells are characteristically oligoclonal and exhibit a differentiated effector memory or terminal effector phenotype (5). As individuals age with CMV, virus-specific cells expand and comprise an increasing fraction of T cells in blood, particularly within the CD8^+^ T cell compartment (5). This phenomenon, known as CD8^+^ memory T cell inflation, constitutes a classical aging-associated immune profile and has been linked with several aging-associated morbidities, including cardiovascular disease, frailty, and neurocognitive impairment (6–8).

More than 90% of people with HIV-1 (PWH) are CMV seropositive (9). Compared with CMV-seropositive individuals without HIV-1, CMV-specific CD4^+^ and CD8^+^ T cells are elevated in the circulation of PWH (on par with levels observed in elderly populations) and remain elevated even with HIV-1 suppression by long-term antiretroviral therapy (ART) (5, 10, 11). In PWH, the clonal expansion of CMV-specific T cells is believed to contribute to cardiovascular complications and support the persistence of the HIV-1 reservoir (12–16). Asymptomatic CMV infection may also shape the immune system more broadly. For example, systems-based approaches in healthy CMV-discordant twins revealed that out of 204 innate and adaptive immune measurements, 119 (58%) were influenced by CMV serotype (17). Furthermore, blockade of asymptomatic CMV replication with the antiviral drug letermovir in ART-suppressed PWH was recently shown to induce broad declines in a number of inflammatory and cardiometabolic plasma proteins (18). While these data suggest the influence of CMV by mechanisms that go beyond the clonal expansion of CMV-specific cells, how the unique immunological environment imparted by CMV may influence early pathological events in HIV-1/SIV infection is not clear.

The high seroprevalence of CMV in PWH is a major obstacle to determining causal relationships in this regard. Previous studies in PWH have stratified CMV-seropositive cohorts by the presence or absence of CMV shedding in genital secretions (19, 20). Subclinical CMV shedding is largely intermittent, however, and may not fully capture the true lack of a CMV-driven immunological footprint in non-shedders. In this study, we employed a likely unique cohort of rhesus macaques (RMs) that were raised in expanded specific pathogen–free (eSPF) environments, devoid of rhesus CMV (RhCMV) and other persistent viruses throughout life. Relative to these RhCMV^–^ RMs, we observed that target CCR5^+^CD4^+^ T cells in lymphoid tissues were highly expanded in RhCMV^+^ SPF RMs prior to SIV. Most of these cells were CXCR3 expressing and by multiple assays did not exhibit specificity to RhCMV. Upon infection with SIV, RhCMV^+^ animals exhibited higher acute phase seeding of SIV DNA in the gut and a more pronounced CXCR3/CXCL9 chemotactic axis. The data suggest that RhCMV can influence SIV susceptibility and inflammatory homing potential of CD4^+^ T cells beyond those that are specific for RhCMV.

## Results

### RhCMV^+^ animals have naturally higher percentages of CCR5^+^ Th1-like memory CD4^+^ T cells in lymphoid tissues.

To characterize phenotypic differences in lymphocyte populations associated with differential RhCMV infection, tissue samples from blood, peripheral lymph nodes (LN), bone marrow (BM), duodenum, and colon were biopsied in 8 RhCMV^–^ and 12 RhCMV^+^ healthy adult male and female rhesus macaques (Table 1). RhCMV^+^ animals in this study were derived from an outdoor colony in which CMV seroprevalence is endemic, acquired across mucosal surfaces within the first year of life by horizontal transfer of the virus through bodily fluids (21, 22). RhCMV^–^ animals were derived from a specialized colony that were nursery-reared or born to RhCMV^–^ mothers and remained seronegative for RhCMV and other persistent viruses throughout life (commonly referred to as eSPF colonies; Table 1). Both conventional and eSPF colonies at the Tulane National Primate Research Center (TNPRC) were housed throughout life in outdoor enclosures and in close proximity to one another, which is presumed to rule out differing exposure to environmental antigens. Seroprevalence of rhesus lymphocryptovirus (RhLCV; macacine γ-herpesvirus 4), a persistent γ-herpesvirus that is highly homologous to human Epstein-Barr virus (EBV) (23), was variable among RhCMV^+^ and RhCMV^–^ groups (Table 1).

**Figure 1:**
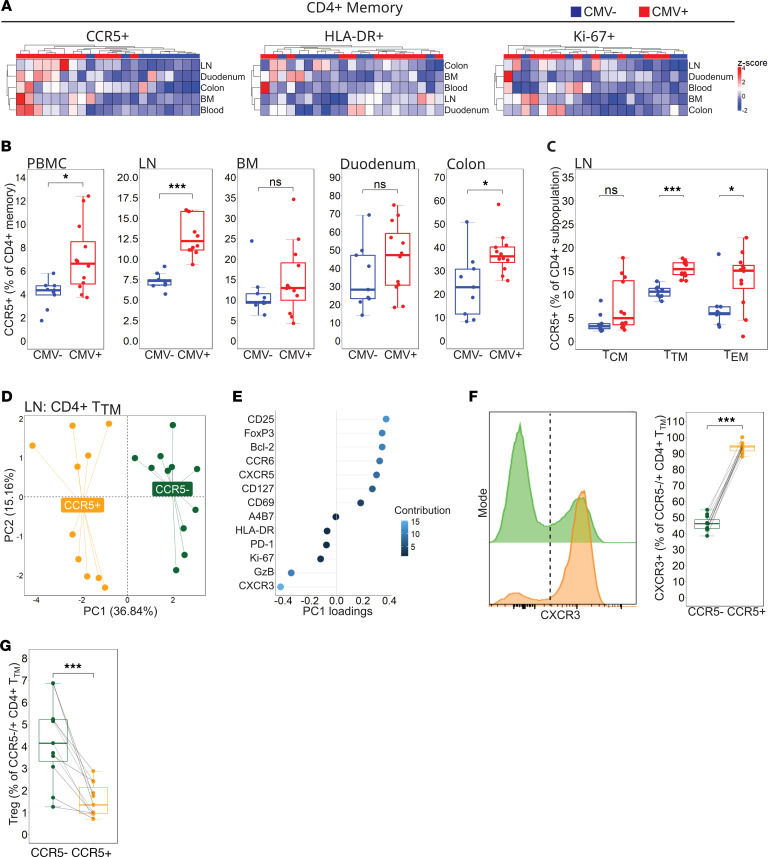
RhCMV infection is associated with increased CCR5 expression and phenotypic remodeling of CD4^+^ memory T cells across tissues pre-SIV. (**A**) Heatmaps with unsupervised clustering by RhCMV^–^ serostatus on %CCR5/HLA-DR/Ki-67 CD4^+^ memory T cells across sampled tissues (RhCMV^+^ *n* = 12; RhCMV^–^ *n* = 9). (**B**) %CCR5^+^CD4^+^ memory box plots in each sampled tissue (RhCMV^+^ *n* = 12; RhCMV^–^ *n* = 9). (**C**) %CCR5^+^ across CD4^+^ T_CM_, T_TM_, and T_EM_ subpopulations (RhCMV^+^ *n* = 12; RhCMV^–^ *n* = 9). (**D**) PCA plot of CCR5^+^ and CCR5^–^ RhCMV^+^CD4^+^ T_TM_ cells in the RhCMV^+^ LN (*n* = 11). (**E**) PC1 loadings with color-coded contribution percentage for the PCA plot (*n* = 11). (**F**) Histogram and %CXCR3^+^ between CCR5^+^/^–^ CD4^+^ T_TM_ in RhCMV^+^ LN (*n* = 11). (**G**) %Treg between CCR5^+^/^–^ CD4^+^ T_TM_ in RhCMV^+^ LN (*n* = 11). Error bars represent 1.5 times the interquartile range. For **B** and **C** statistical comparisons performed using 2-sided Mann-Whitney *U* test. For **F** and **G** statistical comparisons performed using 2-sided Wilcoxon matched pairs signed-rank test. PBMC, peripheral blood mononuclear cells; LN, lymph node; BM, bone marrow; T_CM_, central memory; T_TM_, transitional memory; T_EM_, effector memory. * *P* < 0.05; *** *P* < 0.001.

We first confirmed the lack of an RhCMV-driven immunological footprint in eSPF animals as defined by the degree of effector memory CD4^+^ and CD8^+^ T cell expansion in blood (gating strategy, Supplemental Figure 1; supplemental material available online with this article; https://doi.org/10.1172/jci.insight.198743DS1). RhCMV^+^ animals exhibited comparable numbers of naive (T_N_), central (T_CM_), and transitional memory T (T_TM_) cells in blood, to RhCMV^–^ animals; however, CD4^+^ and CD8^+^ effector memory T (T_EM_) cells were highly expanded (Supplemental Figure 2, A and B). These differences were not observed when animals were stratified by RhLCV serostatus (Supplemental Figure 2, C and D) or sex (Supplemental Figure 2, E and F). We next assessed whether RhCMV was associated with particular CD4^+^ T cell activation states known to influence HIV-1/SIV permissiveness and productive infection. We defined expression of 3 phenotypic markers on total CD95^+^ memory CD4^+^ T cells, including the HIV-1/SIV co-receptor CCR5, activation marker HLA-DR, and cell cycling marker Ki-67, and performed hierarchal clustering of these markers across tissues. We detected no discernable pattern of HLA-DR and Ki-67 expression that distinguished these markers by RhCMV serostatus (Figure 1A). Memory CD4^+^ T cell CCR5 expression, however, clustered highly by serostatus, with only 2 of the total 21 animals that did not stratify by RhCMV serotype (Figure 1A).

Frequencies of CCR5^+^ memory CD4^+^ T cells within the blood, the colon, and especially the LN were significantly higher in RhCMV^+^ animals (Figure 1B), but not when animals were stratified by RhLCV serotype (Supplemental Figure 3A) or sex (Supplemental Figure 3B). These differences were also noted on memory CD8^+^ T cells within the LN and duodenum of RhCMV^+^ (Supplemental Figure 3C) but not in RhLCV^+^ animals (Supplemental Figure 3D) or when segregated by sex (Supplemental Figure 3E). In addition, the MFI of CCR5 in CD4^+^CCR5^+^ cells was elevated in the blood, LN, and BM of CMV-seropositive animals, indicating that these animals exhibited higher per-cell CCR5 expression compared with CMV-seronegative controls (Supplemental Figure 3F). Plasma levels of 2 CCR5 ligands, CCL3 and CCL4, which directly bind and internalize the CCR5 receptor, were similar among RhCMV^–^ and RhCMV^+^ groups (Supplemental Figure 3, G and H) (24, 25), suggesting that RhCMV-associated increases in CCR5 surface density directly ex vivo were not due to altered ligand-receptor occupancy. We also assessed CCR5 surface expression on memory CD4^+^ T cell phenotypes in a human cohort of human CMV (HCMV) -seropositive and -seronegative adults without HIV-1. Surface expression of CCR5 was upregulated on terminal effector memory CD4^+^ T (T_EMRA_) cells in blood (Supplemental Figure 3I), suggesting similar immunologic impact between rhesus and human CMV.

Because the total CD95^+^ memory CD4^+^ T cell pool in RMs contains subpopulations of varying maturation, we measured CCR5 expression on distinct maturation subsets (T_CM_, T_TM_, and T_EM_). In the RhCMV^+^ LN where CCR5 expression was most starkly enriched, expression of CCR5 was found to be significantly higher in T_TM_ and, to a lesser degree, T_EM_ CD4^+^ T cells, with no differences in the CD4^+^ T_CM_ subpopulation (Figure 1C). CCR5^+^CD4^+^ T_TM_ cells in the LN of RhCMV^+^ animals were phenotypically distinct from their CCR5^–^ counterparts and demonstrated complete separation by principal component analysis (PCA) when characterized by an array of phenotypic markers related to lineage, survival, migration, activation, and exhaustion (Figure 1, D and E; gating strategy, Supplemental Figure 4). The phenotypic marker that most strongly segregated CCR5^+^ from CCR5^–^CD4^+^ T_TM_ cells was CXCR3, a chemokine receptor enriched on IFN-γ–producing type 1 helper (Th1) CD4^+^ T cells (Figure 1E) (26, 27). Indeed, CCR5^+^CD4^+^ T_TM_ cells in RhCMV^+^ animals were near-uniformly positive for CXCR3 surface expression (Figure 1F). Conversely, expression of the markers CD25 and FoxP3 that define a T regulatory phenotype were the most distinguishing features in CCR5^–^D4^+^ T_TM_ cells (Figure 1, E and G).

### CCR5^+^CD4^+^ memory T cells expanded in RhCMV^+^ animals are polyclonal, and the majority do not react to an RhCMV lysate.

**Figure 2:**
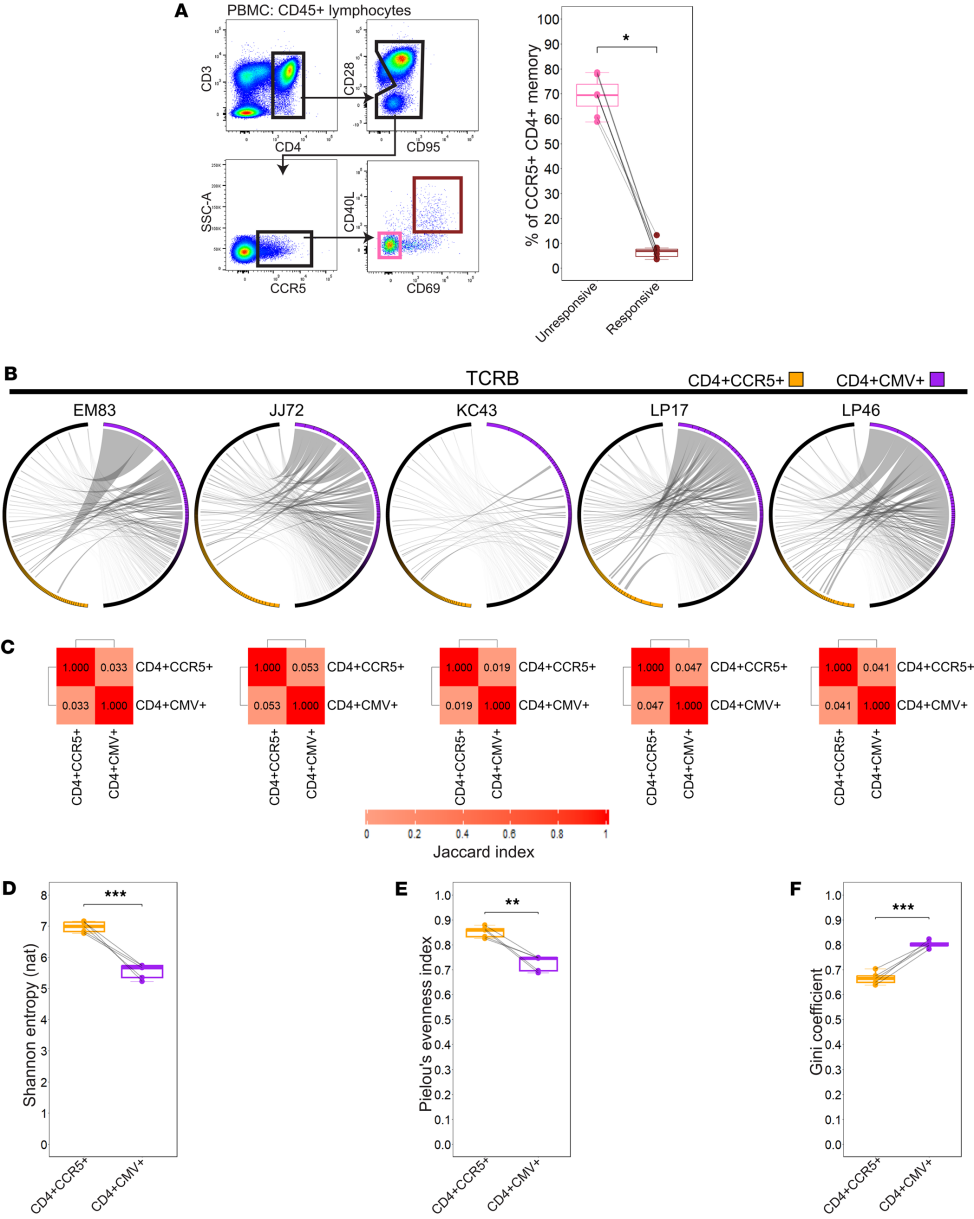
CD4^+^CCR5^+^ T cells exhibit high clonal diversity and limited overlap with CD4^+^ CMV-specific T cells pre-SIV. (**A**) Activation-induced marker assay gating strategy and %CCR5^+^CD4^+^ memory T cells RhCMV specificity box plot based on CD40L and CD69 expression (*n* = 7). The gate in the CD95/CD28 biplot was drawn to exclude CD95^–^CD28^+^ naive CD4^+^ T cells. (**B**) Circos plots where each segment around the circle represents a unique T cell receptor-β (TCRB) complementarity-determining region 3 amino acid (CDR3aa) sequence identified between CD4^+^CCR5^+^ and CD4^+^CMV-specific (CD4^+^CMV^+^) groups. Ribbons connecting segments indicate shared CDR3aa sequences, illustrating the extent of clonal overlap between the 2 groups. The most expanded clones appear in bright orange or purple, while progressively smaller clones are shaded darker, culminating in black for the least abundant clonotypes (*n* = 5). (**C**) Heatmap displaying the Jaccard index of CDR3aa overlap between CD4^+^CCR5^+^ and CD4^+^CMV^+^ per animal (*n* = 5). (**D**) Shannon entropy based on natural logarithm (nat) box plots for each paired sample (*n* = 5). (**E**) Pielou’s evenness index box plots for each paired sample (*n* = 5). (**F**) Gini coefficient box plots for each paired sample (*n* = 5). For **A** and **D**–**F** error bars represent 1.5 times the interquartile range. For **A** statistical comparisons performed using 2-sided Wilcoxon matched pairs signed-rank test. For **D**–**F** statistical comparisons performed using 2-sided paired *t* test. PBMC, peripheral blood mononuclear cells; * *P* < 0.05; ** *P* < 0.01; *** *P* < 0.001.

Because CMV-specific CD4^+^ T cells exhibit canonical Th1 gene signatures (28–30), and CCR5 is a surface marker of Th1 cells (31), we asked whether RhCMV-specific cells could explain the expansion of CCR5^+^CD4^+^ T cells in the RhCMV^+^ LN. We first measured memory CCR5^+^CD4^+^ T cell reactivity to an RhCMV lysate ex vivo by the activation-induced markers (32). To confirm specificity, we assessed the expression of these markers under unstimulated conditions, where significantly fewer reacting CD4^+^ T cells were observed relative to RhCMV lysate exposure (Supplemental Figure 5). In PBMCs from RhCMV^+^ animals, the majority of CCR5^+^ memory CD4^+^ T cells did not respond to an RhCMV lysate (Figure 2A), pointing to specificity beyond RhCMV antigens.

To probe reactivity of the CD4^+^CCR5^+^ T cell pool in more granular detail, we independently sorted resting CCR5-expressing CD4^+^ T cells and CD4^+^ T cells that were exposed ex vivo to the RhCMV lysate and performed bulk RNA sequencing of the CDR3 variable region of *TRA* and *TRB* genes from 5 healthy RhCMV^+^ animals. Ranking the abundance of *TRB* clonotypes and assessing overlap between CMV-responding and resting CCR5^+^CD4^+^ T clones revealed that many CMV-responding clonotypes were shared within the repertoire of the CCR5-expressing pool (Figure 2B), suggesting that the majority of CMV-specific CD4^+^ T cells express CCR5 on the cell surface at resting states in vivo. Conversely, only a small minority of resting CCR5^+^ clonotypes were shared among clones that responded to the RhCMV lysate (Figure 2B). Shared clonotypes were interspersed among the rank order of CCR5^+^ clonotypes, and rarely present in high abundance (Figure 2B). To quantify the representation of CMV-reactive clones within the CCR5-expressing repertoire, we calculated the Jaccard index of the 2 sorted populations, where a value of 1 equals identical repertoires, and 0 equals repertoires that are completely unique from one another. This revealed among all animals that the repertoires between populations were largely distinct and non-overlapping (Figure 2C). Furthermore, diversity metrics indicated that distinct clonotypes were less dominant within the repertoire of resting CCR5^+^CD4^+^ T cells when compared with those of CMV-responding cells (Shannon diversity, Pielou’s evenness; Figure 2, D and E), whereas CMV-responding cells were more clonally skewed (Gini coefficient; Figure 2F). Taken together, the data indicate that RhCMV expands CCR5^+^CD4^+^ T cells beyond those that are specific for RhCMV antigens.

### Enhanced seeding of SIV DNA in the RhCMV^+^ gut during acute SIV.

**Figure 3:**
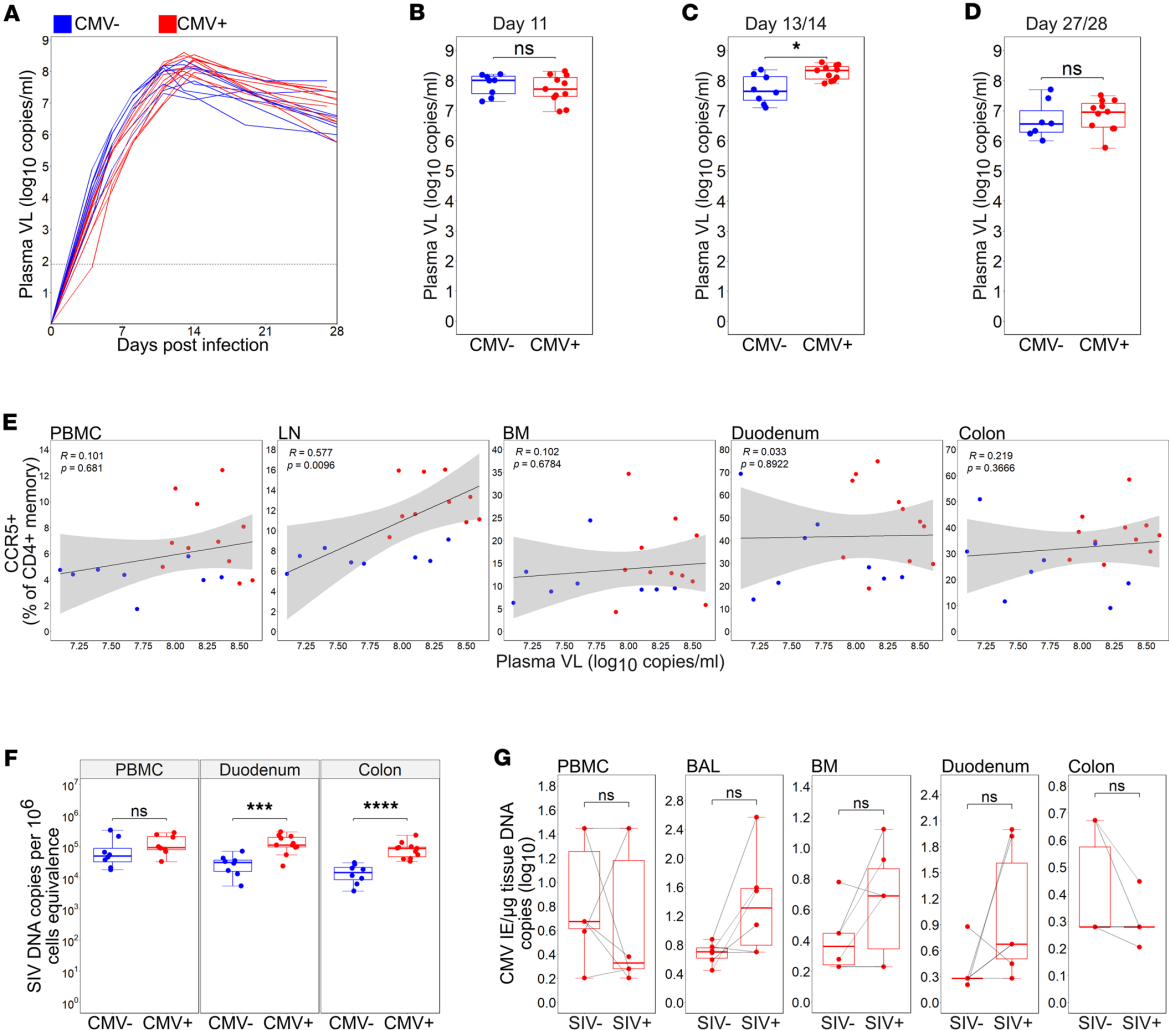
RhCMV serostatus is associated with increased SIV replication. (**A**) Line graph of plasma SIV viral loads (VL) over days after infection. (**B**–**D**) Plasma VL on day 11, day 13/14, and day 27/28 by RhCMV serostatus. (**E**) Two-sided Spearman’s correlation between pre-SIV %CCR5^+^CD4^+^ memory against plasma VL on day 13/14. Shaded area represents 95% confidence interval. (**F**) Cell-associated SIV DNA metrics by RhCMV serostatus. (**G**) The number of CMV copies per microgram of tissue DNA between uninfected (SIV^–^) and day 13/14 SIV-infected (SIV^+^) animals (SIV^–^ *n* = 6; SIV^+^ *n* = 6). For **A**–**F** RhCMV^+^ *n* = 11; RhCMV^–^ *n* = 8, statistical comparison performed using 2-sided Mann-Whitney *U* test. For **G** statistical comparisons performed using 2-sided Wilcoxon matched pairs signed-rank test. For **B**–**D**, **F**, and **G** error bars represent 1.5 times the interquartile range. LN, lymph node; BM, bone marrow; PBMC, peripheral blood mononuclear cells; BAL, bronchoalveolar lavage; * *P* < 0.05; *** *P* < 0.001; **** *P* < 0.0001.

In both CD4^+^ and CD8^+^ T cells, molecules that distinguish a Th1 program are commonly associated with more effective viremic control of HIV (33–35). On the other hand, Th1 cells are readily infected by HIV-1/SIV and can harbor large clonally expanded populations of HIV-1 proviruses during ART (36, 37). To determine the relative benefit or detriment of an RhCMV-driven, Th1-skewed environment on acute SIV replication dynamics, we infected study animals intravenously with barcoded SIV239M and sampled whole blood at time windows of exponential SIV growth (4, 6, 8 days postinfection; dpi), peak viremia (11, 13/14 dpi), and post-peak SIV decline (21, 28 dpi; Figure 3A). Within time windows of peak SIV replication, there were no significant differences in viremia among RhCMV^–^ and RhCMV^+^ animals at 11 dpi (Figure 3B). However, at 13/14 dpi, plasma SIV load was higher in co-infected animals (Figure 3C). SIV viral load at 13/14 dpi was not associated with RhLCV status (Supplemental Figure 6A) or sex (Supplemental Figure 6B). Elevated viremia in this group was transient as both groups exhibited similar viral loads after post-peak SIV decline at 28 dpi (Figure 3D). Only in the LN did we observe a direct association between peak viremia and pre-infection frequencies of CCR5^+^CD4^+^ T cells (Figure 3E), suggesting that out of all anatomic sites, target cell densities in lymphoid tissues were the most determinative of peak viral loads.

We next determined the levels of total cell-associated SIV DNA in PBMCs and gut mucosal tissues at 14 dpi. There were no significant differences in the frequencies of cells harboring SIV DNA within the blood of RhCMV^–^ and RhCMV^+^ animals (Figure 3F). In duodenal and colonic tissues, however, total SIV DNA levels were higher at peak viremia in RhCMV^+^ (Figure 3F) but not RhLCV^+^ RMs (Supplemental Figure 6C) or when segregated by sex (Supplemental Figure 6D). The RhCMV^+^ colon in particular exhibited median SIV DNA levels that were roughly log_10_ 0.8 (6.3-fold) higher than colonic tissue of the RhCMV^–^ group (RhCMV^+^: 4.94; RhCMV^–^: 4.18; Figure 3F). We next considered the possibility that the higher SIV burden in the RhCMV^+^ gut was due to the fact that the GI tract was a preferential site of RhCMV reactivation during acute SIV. In a subset of co-infected animals, we assessed levels of RhCMV DNA prior to SIV and at peak SIV viremia across anatomic sites. However, we did not observe acute SIV to induce discernable patterns in RhCMV reactivation rates that were preferential to a particular tissue site and notably the gut mucosa (Figure 3G). Taken together, the data suggest that RhCMV promotes seeding of the SIV reservoir in a site-specific manner but that this is not necessarily related to a shared tissue tropism of the 2 co-infecting viruses.

### Limited CD8^+^ T cell cycling and enhanced maintenance of CCR5^+^CD4^+^ T cells in the RhCMV^+^ gut during acute SIV.

**Figure 4:**
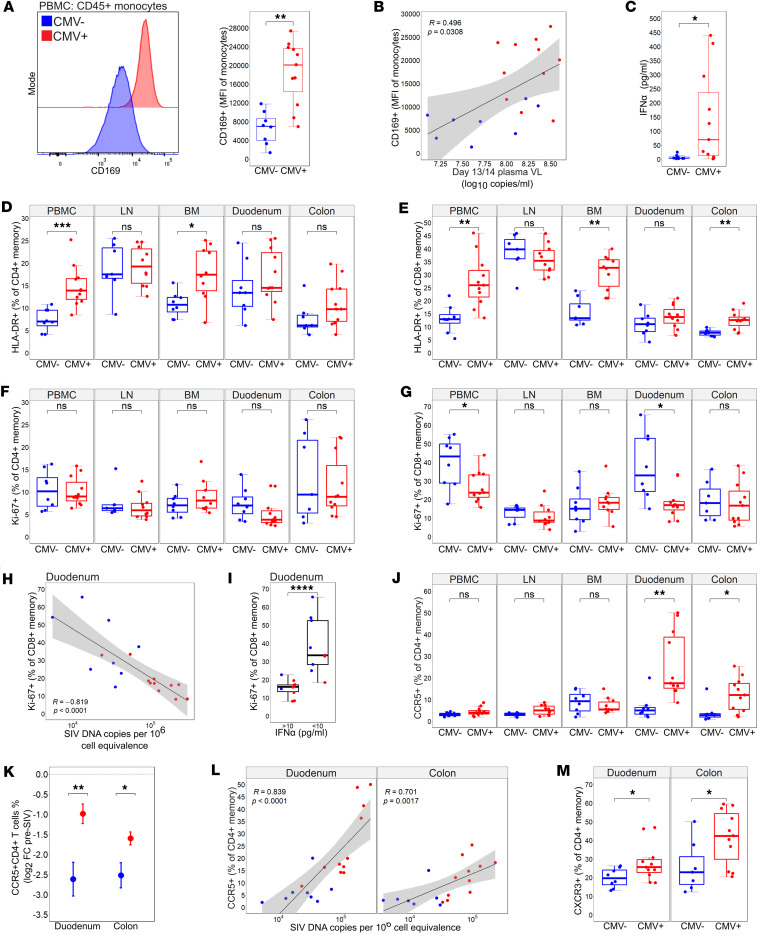
RhCMV serostatus during SIV infection is associated with heightened monocyte activation and increased persistence of CCR5^+^CD4^+^ T cells in the gut. (**A**) Histogram and CD169^+^ monocyte median fluorescence intensity (MFI) by RhCMV serostatus in PBMC. (**B**) Two-sided Spearman’s correlation between CD169^+^ monocyte MFI against plasma VL. (**C**) Plasma IFN-α expression by RhCMV serostatus. (**D**) %HLA-DR^+^CD4^+^ memory T cells across sampled tissue by RhCMV serostatus. (**E**) %HLA-DR^+^CD8^+^ memory T cells across sampled tissue by RhCMV serostatus. (**F**) %Ki-67^+^CD4^+^ memory T cells across sampled tissue by RhCMV serostatus. (**G**) %Ki-67^+^CD8^+^ memory T cells across sampled tissue by RhCMV serostatus. (**H**) Two-sided Spearman’s correlation between %Ki-67^+^CD8^+^ memory T cells against cell-associated SIV DNA. (**I**) %Ki-67^+^CD8^+^ memory T cells by plasma IFN-α above or below 10 pg/mL (>10 and <10 respectively). (**J**) %CCR5^+^CD4^+^ memory T cells across sampled tissue by RhCMV serostatus. (**K**) %CCR5^+^CD4^+^ T cells log_2_ fold change (FC) pre-SIV mean ± SEM across duodenum and colon by RhCMV serostatus. (**L**) Two-sided Spearman’s correlation between %CCR5^+^CD4^+^ memory cells against cell-associated SIV DNA. (**M**) %CXCR3^+^ of CD4^+^ memory T cells across duodenum and colon by RhCMV serostatus. For all figures RhCMV^+^ *n* = 11, RhCMV^–^ *n* = 8. For **A**, **B**, **D**–**G**, **I**, **J**, and **M** error bars represent 1.5 times the interquartile range. Statistical comparison performed using 2-sided Mann-Whitney *U* test. For **B**, **H**, and **L** shaded area represents 95% confidence interval. LN, lymph node; BM, bone marrow; PBMC, peripheral blood mononuclear cells; * *P* < 0.05; ** *P* < 0.01; *** *P* < 0.001; **** *P* < 0.0001.

We next sought to determine how RhCMV infection influenced the acute SIV dynamics of innate and adaptive immune cells. We first probed how co-infection influenced phenotypic profiles of circulating monocytes at 13/14 dpi with SIV. The type I IFN–stimulated surface marker CD169 was found to be elevated on monocytes of RhCMV^+^ animals and correlated directly with viremia at 13/14 dpi (Figure 4, A and B). We also measured systemic levels of IFN-α in plasma at this period by conventional ELISA. Co-infected animals exhibited higher plasma concentrations of IFN-α at peak viremia (Figure 4C), together indicating that RhCMV enhanced the type I IFN response during acute SIV.

Co-infection with RhCMV did not impact the degree of CD4^+^ T cell depletion across tissues during acute SIV, although both study groups exhibited a precipitous drop in CD4^+^ T cell frequencies at gut mucosal tissues in line with early target cell depletion at these sites (Supplemental Figure 7A) (38, 39). Surface expression of the activation marker HLA-DR was higher on CD4^+^ and CD8^+^ memory T cells in the BM of co-infected animals at 13/14 dpi but not within the colon and duodenum (Figure 4, D and E). HLA-DR surface expression was also highly increased on circulating CD4^+^ and CD8^+^ memory T cells of RhCMV^+^ animals during acute SIV (Figure 4, D and E), although this was likely related in large part to the expansion of HLA-DR^+^ T_EM_ cells in blood prior to SIV infection (Supplemental Figure 7, B and C), consistent with the chronic immune activation characteristic of CMV^+^ animals. We did not observe the frequencies of Ki-67^+^ cycling memory CD4^+^ T cells to differ across tissues by RhCMV serostatus at 13/14 dpi (Figure 4F). However, co-infected animals exhibited significantly reduced cycling of memory CD8^+^ T cells in the blood and duodenum during acute SIV (Figure 4G), which in the duodenum correlated inversely with levels of SIV DNA (Figure 4H). Type I IFNs upregulate several proteins that induce G1 cell cycle arrest (40–42). To probe the relationship between IFN-α and the limited cycling of memory CD8^+^ T cells in co-infected animals during acute SIV, we binned all study animals by low (<10 pg/mL) or high (>10 pg/mL) IFN-α plasma concentrations at 13/14 dpi and compared the degree of duodenum memory CD8^+^ T cell cycling between the 2 bins. We observed that in animals with high concentrations of plasma IFN-α, cycling memory CD8^+^ T cells in the duodenum were significantly lower relative to that of the IFN-α–low group (Figure 4I), suggesting that in co-infected animals the enhanced type I IFN response limited CD8^+^ T cell proliferation at prominent early sites of SIV replication.

We last assessed CCR5^+^ target cells across tissues during acute SIV and how their frequencies were influenced by RhCMV coinfection. CCR5^+^ memory CD4^+^ T cells within blood and LNs were equally reduced among RhCMV^–^ and RhCMV^+^ animals at 13/14 days post-SIV infection (Figure 4J). At gut mucosal sites, however, RhCMV^+^ animals continued to exhibit expanded target cell frequencies in the colon during acute SIV (Figure 4J). Frequencies of target cells were also elevated in the duodenum of coinfected animals (Figure 4J), a feature that was not observed at this site prior to SIV (Figure 1B). Together, the maintenance of CCR5^+^CD4^+^ T cells in the RhCMV^+^ gut led to a significantly smaller fold change in target cell depletion within the colon and duodenum (Figure 4K) and was associated with higher levels of SIV DNA in the gut (Figure 4L).

We sought to determine potential mechanisms underlying the apparent discordant finding of greater SIV DNA seeding, yet less target cell reduction in the RhCMV^+^ gut. We first considered the possibility that mucosal resident memory CD4^+^ T cells of RhCMV^+^ animals exhibited superior survival capabilities that would allow them to withstand cell death during SIV infection. We examined expression of the interleukin-7 receptor CD127 and anti-apoptotic molecule Bcl-2, known to promote T cell survival (43, 44), yet found no differences in memory CD4^+^ T cell expression of these markers at gut mucosal sites during acute SIV by RhCMV serostatus (Supplemental Figure 8, A and B). We next considered the possibility that CCR5^+^ target cell depletion could be offset by a greater rate of influx of memory CD4^+^ T cells into the RhCMV^+^ gut during acute SIV. We assessed surface expression levels of the chemokine receptor CXCR3, because ligands of this receptor (CXCL9–11) are IFN inducible and highly upregulated during viral infection, and expanded CCR5^+^CD4^+^ T cells expanded uniformly expressed CXCR3 (Figure 1F). Indeed, frequencies of CXCR3-expressing memory CD4^+^ T cells were increased in the RhCMV^+^ gut (Figure 4M), despite exhibiting comparable frequencies to those of RhCMV^–^ animals prior to SIV infection (Supplemental Figure 8C). Taken together, the data suggest that RhCMV can influence the trafficking potential of CD4^+^CCR5^+^ T cells and promote the maintenance of target cells at early sites of SIV replication.

### RhCMV enhances the CXCL9/CXCR3 chemokine axis before and after SIV infection.

**Figure 5:**
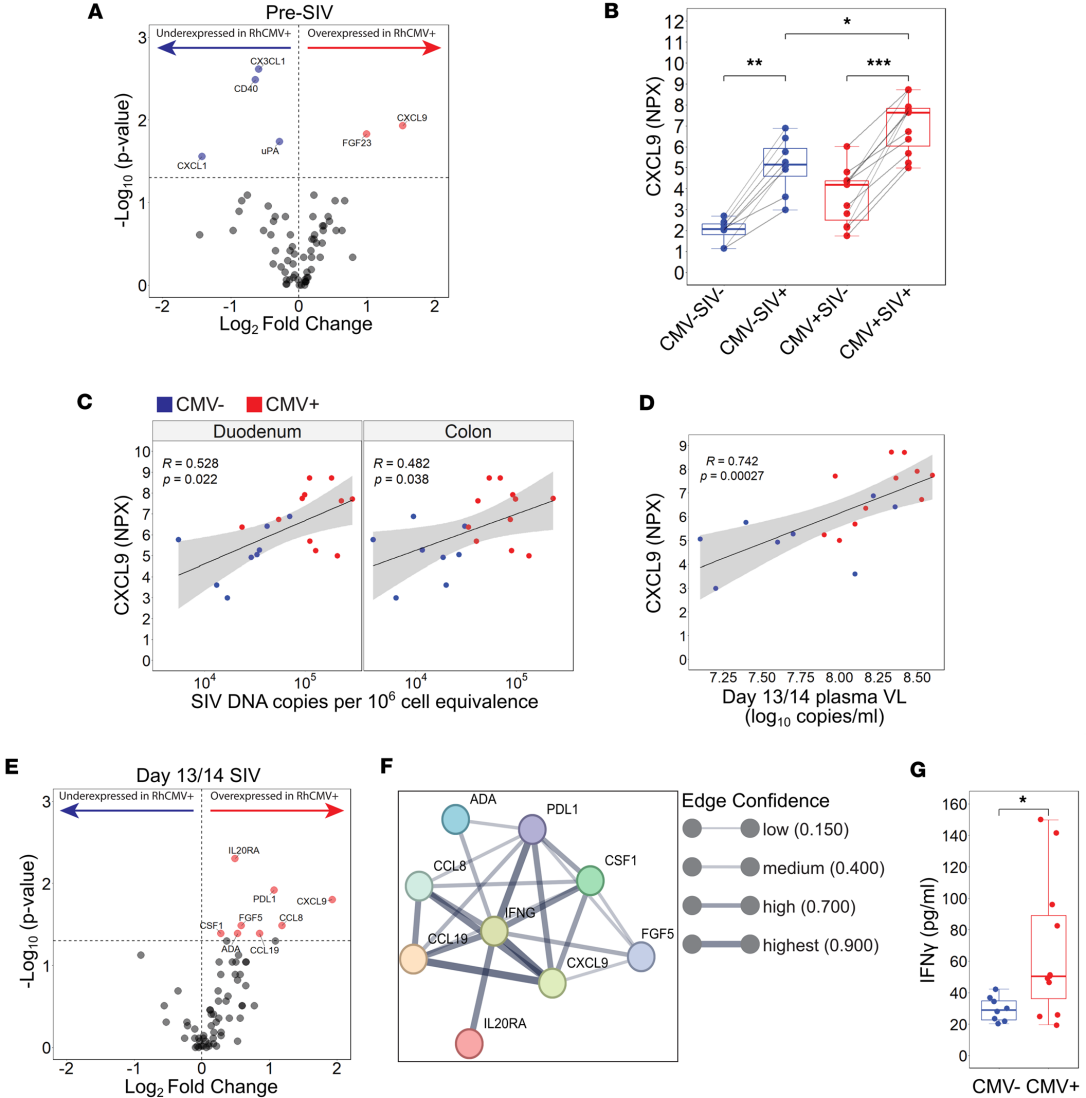
RhCMV serostatus modulates IFN-γ–associated chemokine signatures correlating with SIV burden. (**A**) Volcano plot representing the mean log_2_ fold change in plasma chemokines found in SIV-uninfected animals based on RhCMV serostatus. Red overexpressed; blue underexpressed; gray nonsignificant. (**B**) CXCL9 plasma expression between SIV serostatus and RhCMV serostatus. (**C**) Two-sided Spearman’s correlation between post-SIV plasma CXCL9 against plasma SIV VL. (**D**) Two-sided Spearman’s correlation between post-SIV plasma CXCL9 against cell-associated SIV DNA across duodenum and colon. (**E**) Volcano plot representing the mean log_2_ fold change in plasma chemokines found in day 13/14 dpi SIV-infected animals based on RhCMV serostatus. Red overexpressed; blue underexpressed; gray nonsignificant. (**F**) STRING network representing the interaction of the RhCMV-associated overexpressed proteins with IFN-γ. (**G**) Post-SIV plasma IFN-γ by RhCMV serostatus. For all figures RhCMV^+^ *n* = 11, RhCMV^–^ *n* = 8. For **B** and **G** error bars represent 1.5 times the interquartile range. For **B** statistical comparisons performed using 2-sided Wilcoxon matched pairs signed-rank test, between SIV^–^ and SIV^+^, and 2-sided Mann-Whitney *U* test between RhCMV^–^ and RhCMV^+^. For **A**, **E**, and **G** statistical comparison performed using 2-sided Mann-Whitney *U* test. For **C** and **D** the shaded area represents 95% confidence interval. NPX, normalized protein expression; * *P* < 0.05; ** *P* < 0.01; *** *P* < 0.001.

The ligands for CXCR3 are the IFN-inducible chemokines CXCL9–11, and this axis contributes to the recruitment of immune cells to gut mucosal surfaces during inflammation (45, 46). We thus performed high-throughput proteomic profiling of over 60 inflammatory proteins, including CXCR3 ligands, in plasma by proximity extension assay to assess cytokine/chemokine profiles by RhCMV serotype. In healthy animals prior to SIV infection, asymptomatic RhCMV infection in general exerted a relatively muted impact on most of the inflammatory analytes, with soluble mediators of neutrophil chemotaxis (CXCL1), the co-stimulatory molecule CD40, and the chemotactic/adhesive molecule sCX3CL1 as factors being underexpressed in RhCMV (Figure 5A). The soluble protein most significantly differentially upregulated in plasma of SIV-uninfected RhCMV^+^ animals was the CXCR3 ligand CXCL9 (Figure 5A).

To verify this finding, we further assessed CXCL9 plasma concentrations in a larger cohort of SIV-uninfected RhCMV^+^ RMs that exhibited varying frequencies of CD8^+^ T_EM_ expansion in blood, the hallmark immunological footprint of CMV. The chemokines CXCL10 and CXCL11 were also examined, which are coordinately regulated with CXCL9 and are additional ligands for CXCR3. We then stratified CD8^+^ T_EM_ frequencies in RhCMV^+^ RMs by quartile, with Q1 being the lowest and Q4 the highest (Supplemental Figure 9A). Both plasma CXCL9 and CXCL10 levels were highest among RhCMV^+^ animals falling within the quartile of greatest CD8^+^ T_EM_ expansion (Q4; Supplemental Figure 9, B and C). CXCL11 levels across quartiles did not reach statistical significance based on the Kruskal-Wallis test; however, a post hoc analysis revealed a significant difference between the lowest and highest quartiles (Supplemental Figure 9D).

In RMs that went on to be experimentally infected with SIV, CXCL9 plasma concentrations increased in all RMs regardless of CMV serotype yet remained elevated in RhCMV^+^ animals at 14 dpi (Figure 5B). We observed significant, direct relationships between CXCL9 levels in plasma and both SIV DNA copies in the gut and viremia at 13/14 dpi (Figure 5, C and D). Furthermore, CXCL9 elevations existed within a larger network of soluble proteins that were differentially overexpressed in co-infected animals, including sPDL1, CSF1, CCL19, and the CCR2/CCR5 ligand CCL8 (Figure 5E). We next performed STRING analysis to infer interactions among overexpressed proteins in RhCMV animals during acute SIV. Many of these proteins were found to exhibit interconnectivity with high confidence as defined by predicted/known protein-protein interaction (Figure 5F). Of note, the canonical Th1 cytokine IFN-γ was predicted to occupy a central node within this network (Figure 5F). Measuring IFN-γ levels by ELISA revealed that IFN-γ was indeed systemically higher in co-infected animals at 13/14 dpi (Figure 5G). Overall, the cellular and cytokine/chemokine data are consistent with RhCMV driving a global IFN-γ–directed Th1 signature, which becomes more pronounced during acute SIV and is associated with the maintenance of target cells and enhanced seeding of SIV DNA in gut mucosal tissues.

## Discussion

HIV-1/SIV replication dynamics are critically dependent on the density of CCR5-expressing target cells. In both HIV-1 and SIV, there are natural settings in which the host exhibits exceptionally low densities of target cells, and these instances are associated with either spontaneous viremic control or long-term non-progression (47–50). Here, we describe an apparent contrary setting in which natural chronic asymptomatic infection with the highly prevalent nonhuman primate homolog of human CMV leads to expansion of CCR5-expressing target cells at early sites of viral replication and exacerbates SIV burden. This feature was not observed when stratifying our study animals by RhLCV serostatus. It is also unlikely that these differences were due to disparate environmental factors between the eSPF and conventional colonies, as both are maintained outdoors at the TNPRC and in close proximity to each other. Given the well-documented role of CMV in influencing systemic and tissue microenvironments (51–55), our findings suggest that RhCMV infection can alter the host immune environment in a way that enhances acute SIV replication.

We show here that target cells expanded in RhCMV^+^ animals exhibit distinguishing phenotypic signatures, including a near-uniform CXCR3^+^ Th1-like profile and a predominant transitional memory (T_TM_) maturation phenotype. In virally suppressed PWH on ART, large clonal expansions of intact HIV-1 proviruses in blood can be concentrated in functionally polarized Th1 cells (36). Moreover, the predominant T_TM_ phenotype suggests infection of these cells could result in the seeding of SIV DNA in a maturation subtype that is long-lived, responsive to homeostatic cytokines, and known to contribute to the pool of latent HIV-1 in long-term ART-suppressed persons (56). An additional feature of target cells in RhCMV^+^ animals was that the pool was largely polyclonal and did not exhibit specificity to RhCMV lysate, indicating that RhCMV can expand the pool of SIV-susceptible cells beyond those that are specific for CMV. In further support of this, we observed that target cell expansion was most pronounced in lymphoid tissues. In humans, the majority of CMV-specific T cells, however, are excluded from lymphoid tissues and do not traffic to these sites (57–59).

At present, the precise RhCMV-driven environmental cues that induce and maintain CCR5 expression on memory CD4^+^ T cells are not known. In vitro, it is difficult to induce CCR5 expression in resting CD4^+^ T cells, even by supraphysiologic means with polyclonal activation stimuli (60). CMV has been shown to be able to stimulate CCR5 expression in central memory cord blood mononuclear cells in vitro (61). In both mice and humans, the addition of the Th1-polarizing cytokine IL-12 to culture media in combination with αCD3/αCD28 stimulation can induce the upregulation of CCR5 in activated CD4^+^ T cells (62, 63). Our data are thus consistent with an RhCMV-driven Th1-polarizing environment, which is canonically driven in part by IL-12, as a potential contributor to target cell maintenance. In vivo, the primary sources of IL-12 are dendritic cells within lymphoid tissues (64). Our data are thus also consistent with the lymphoid tropic nature of target cell expansion in RhCMV, which is the main site of direct interaction between T cells and dendritic cells.

We show here that expansion of CXCR3^+^ Th1-like target cells in RhCMV is accompanied by higher expression of the IFN-γ–inducible CXCR3-binding chemokine CXCL9 in plasma. No differences in plasma CXCL9 expression in our study animals were observed when stratifying by RhLCV status. Moreover, we confirm in a separate cohort a significant trend of increasing plasma CXCL9 when RhCMV^+^ animals were stratified by effector CD8^+^ T cell proportions in blood, the canonical signature of chronic CMV. In the confirmatory cohort this extended to other IFN-γ–induced CXCR3-binding chemokines as well, including CXCL10 and CXCL11. The relevance of our findings to humans is supported by the observation that plasma levels of CXCL10 are elevated among ART-suppressed HCMV^+^ PWH compared with HCMV^–^ PWH on ART (11). Additionally, plasma levels of CXCL10 are elevated in lung transplant recipients with detectable HCMV replication (65). Thus, induction of CXCR3-binding chemokines, likely existing within a larger IFN-γ–regulated network, may represent a conserved mechanism by which RhCMV and HCMV influence host immunity.

RhCMV/SIV coinfection was associated with several notable acute phase virological distinctions, including transient elevation of viremia at peak SIV and starkly higher cell-associated SIV (CA-SIV) DNA frequencies in the gut. The gastrointestinal tract is a prominent site of pathology and early HIV-1/SIV replication (38, 39). Lamina propria CD4^+^ T cells are highly permissive to HIV-1/SIV infection, and there is a near-complete loss of CD4^+^ T cells at this site during acute infection (38, 39). In untreated HIV-1/SIV infection viral replication is anatomically dispersed, and an outstanding question is how local HIV-1/SIV replication in the gut contributes to the overall dynamics of acute phase viremia relative to other anatomic sites. In our study, it is interesting to note that at 14 dpi, CA-SIV DNA differed by near log-fold levels across the upper and lower intestine, yet distinctions in plasma viremia were relatively modest between RhCMV^+^ and RhCMV^–^ groups and were not sustained at later time points of acute SIV. Our study did not employ longitudinal sampling of gut mucosal sites; however, the data suggest that at least during peak SIV, local viral replication within the gut is not a major contributor to overall levels of plasma viremia. Supporting this notion is our finding that pre-infection frequencies of target cells in the duodenum and colon did not predict levels of peak viremia. Only in lymphoid tissues did we observe pre-infection target cells to correlate with plasma SIV at 14 dpi. This may suggest that peak viremia may largely be determined by the degree of viral replication in lymphoid tissues, with other anatomical sites representing a minority contribution. This dynamic may also extend to the early emergence of viremia following analytical treatment interruptions of ART, as early recrudescing strains of SIV in plasma can be tracked specifically to CA-SIV clonotypes in lymphoid tissues (66).

What could explain the stark enrichment of CA-SIV DNA in the gut of RhCMV-seropositive animals? We show here that the cycling of CD8^+^ T cells in the duodenum of co-infected animals is limited during acute SIV. Notably, co-infected animals also exhibit elevated plasma levels of IFN-α, a cytokine with well-known antiproliferative properties. Thus, one contributing mechanism could be the suppression of antiviral CD8^+^ T cell by locally high concentrations of IFN-α in the GI tract. A second contributing mechanism could relate to the rate of target cell influx into the gut mucosa during acute SIV. We show here at 14 dpi that despite a generally higher RhCMV-associated SIV burden, frequencies of gut mucosal target cells were less depleted in RhCMV^+^ animals when compared with those of the RhCMV^–^ group. Maintenance of target cells could not be explained by differing phenotypic survival signatures known to promote resistance to HIV-1/SIV cell-mediated death (67, 68), but rather, both plasma CXCL9 levels and mucosal CXCR3^+^CD4^+^ T cell frequencies remained elevated during acute SIV, suggesting replenishment of target cells into the RhCMV^+^ gut along a CXCL9/CXCR3 chemotactic axis. We also found that CCL8 was highly overexpressed in RhCMV^+^ RMs during acute SIV, indicating an additional potential migratory axis as CCL8 can bind and induce the chemotaxis of CCR5^+^ T cells (69).

There are notable limitations to our study. First, we cannot infer which mechanism of target cell migration to the gut is dominant in coinfected animals, as our data suggest the contribution of multiple chemotactic routes. Second, while we highlight an apparent lack of RhLCV-mediated virological and immunological imprint in our study animals, we cannot rule out the potential influence of other persistent viruses. While RhCMV-seronegative eSPF colonies are also seronegative for both simian foamy virus (SFV) and rhesus rhadinovirus, these viruses are endemic in conventionally housed animals with seroprevalence greater than 90% (70, 71). While asymptomatic in healthy captive macaques, both viruses can mediate disease in settings of SIV-induced immunodeficiency, with SFV in particular exacerbating viremia and CD4^+^ T cell decline during late stages of SIV infection (72, 73). Third, it is currently unclear how these findings extend to latent CMV infection in humans. We show here in a small cohort of adults without HIV-1 that CCR5 surface expression is marginally increased on terminally differentiated effector memory (T_EMRA_) CD4^+^ T cells in blood of HCMV-seropositive donors. CD4^+^ T_EMRA_ cells exhibit the shortest half-life of all memory subsets (<3.1 months) (74, 75) and furthermore do not survive for longer than 2 days if productively infected (76, 77). The clinical significance of CD4^+^ T_EMRA_ cells as targets for HIV-1 is thus unclear given that these cells are labile and do not comprise the stable reservoir during long-term ART (78). In addition, we note that human gut and lymphoid tissues, which harbor tissue-resident memory CD4^+^ T cells, may be more relevant sites for HIV-1 infection in the context of latent CMV, highlighting the need for future studies in these compartments. A fourth limitation of our study is that absolute CD4^+^ T cell counts could only be measured in blood, as insufficient cells were recovered from tissues. Consequently, tissue data are reported as percentages, which may be influenced by CD4^+^ T cell depletion during acute SIV infection and should be interpreted accordingly. In our study animals, LNs exhibited the highest increase in CCR5 densities associated with RhCMV. Thus, evaluating target cells in lymphoid tissues of HCMV-seropositive and HCMV-negative individuals poses clinical meaningful implications for HIV-1/CMV coinfection.

## Methods

### Sex as a biological variable.

RMs of both sexes were included in this study. The proportion of females to males in both groups can be found in Table 1. Although the relatively small group sizes limit the ability to rigorously assess sex as a biological variable, we nonetheless examined potential sex-related differences in the main findings of this study.

### Animals and infection procedure.

This study was designed with both groups, RhCMV^–^ and RhCMV^+^, having roughly a similar number of male and female animals to avoid sex as a confounding variable. All Indian-origin RMs (*Macaca mulatta*) involved in the study were bred within the Tulane National Primate Research Center (TNPRC), except 3 (RF73–75) that were bred and transferred from the Oregon National Primate Research Center (ONPRC). All animals were negative for Mamu alleles commonly associated with effective SIV control, specifically Mamu-A*01, Mamu-B*08, and Mamu-B*17, except for 1 RhCMV^–^ (KN94), which was positive for Mamu-B*17. Animals housed in the SPF colony naturally acquire CMV infection by the age of 1 year, whereas those in the eSPF colony remain CMV free. Nine RhCMV^–^ and 12 RhCMV^+^ RMs were examined at baseline, of which 8 RhCMV^–^ and 11 RhCMV^+^ animals were infected with SIV. The animals were intravenously infected with 1 mL of SIVmac239M, provided by AIDS and Cancer Virus Program, Frederick National Laboratory for Cancer Research, Frederick, Maryland, USA, containing a concentration of 5,000 IU/mL. Throughout the infection process, animals were anesthetized using telazol tiletamine hydrochloride and zolazepam hydrochloride (5 to 8 mg/kg intramuscular; tiletamine–zolazepam, Zoetis), along with buprenorphine hydrochloride (0.03 mg/kg) for pain management. From the TNPRC colony animals 35 RhCMV^–^ and 75 RhCMV^+^ were randomly selected during a semi-annual health assessment for an extended blood draw to acquire PBMCs for CMV-specific T cell screening and plasma for Olink proteomics.

### Tissue processing.

PBMCs isolation was performed using Ficoll-Paque PLUS (Cytiva) gradient separation and standard procedures. Lymph nodes, duodenum, and colon pinches were mechanically disrupted using gentleMACS C Tubes and gentleMACS Dissociator (Miltenyi Biotec) and filtered through a 100 μm cell strainer. Bone marrow was enriched for lymphocytes using Ficoll-Paque PLUS gradient separation. Cells were cryopreserved in 10% dimethyl sulfoxide (DMSO; EMD Millipore) in fetal bovine serum (FBS; Corning) and stored in liquid nitrogen.

### Complete blood count.

Hematological analysis was performed at the TNPRC clinical lab. Hematological analysis was performed on whole blood, collected in EDTA tubes, using a Sysmex XN-V-1000 Hematology Analyzer. The complete blood count included absolute quantification of red blood cells, neutrophils, monocytes, lymphocytes, basophils, and eosinophils. Counts of T cell subsets were extrapolated by applying flow cytometry–derived subset percentages to the absolute lymphocyte count.

### SIV plasma viral load quantification.

Blood specimens from SIV-infected RMs were collected in EDTA anticoagulant and centrifuged at 900*g* for 15 minutes. Plasma was aspirated, aliquoted into cryovial tubes, and stored at –80°C. SIV plasma viral loads were quantified by TNPRC’s Center Pathogen Detection Core (PDQC) as previously described (79). Briefly, SIV target cDNA and exogenous control cDNA were assayed in duplicate. QuantStudio 12k Flex (Thermo Fisher Scientific) was used with a program of 40 cycles at 95°C for 15 s and 60°C for 1 min. The following primers and probe for SIVmac239 were used: Forward 5′ - AGGCTGCAGATTGGGACTTG - 3′, Reverse 5′ - TGATCCTGACGGCTCCCTAA - 3′, and Probe 5′ - FAM-ACCCACAACCAGCTCCACAACAAGGAC-IABKFQ - 3′.

### CA-SIV DNA quantification.

CD4^+^ T cells were immuno-magnetically separated from bulk mononuclear cell suspensions by positive selection using the CD4^+^ T cell isolation kit, non-human primate, as per the manufacturer’s instructions (Miltenyi Biotec; catalog 130-092-144). Separated CD4^+^ T cells or colonic cell suspensions were lysed with Buffer RLT Plus (QIAGEN; catalog 1053393) and 10% 2-mercaptoethanol (MilliporeSigma; catalog M6250) and DNA was subsequently purified using the AllPrep DNA/RNA kit (QIAGEN; catalog 80204). The CA-SIV DNA quantification was performed employing a standard real-time quantitative PCR protocol with TaqMan Fast Advanced Master Mix (Applied Biosystems; catalog 4444557). The PCR conditions included an initial step of 95°C for 2 min for template denaturation, followed by 40 cycles of 95°C for 30 s and 60°C for 60 s. The primers (and their respective reaction concentrations) used were as previously published and as follows (80): *sGAGF* 5′-GTCTGCGTCATCTGGTGCATTC-3′ (600 nM), *sGAGR* 5′-CACTAGGTGTCTCTGCACTATCTGTTTTG-3′ (600 nM), *sGAGPr* 5′-FAM-CTTCCTCAGTKTGTGTTTCACTTTCTCTTCTGCG-BHQ1-3′ (100 nM), *CCR5F* 5′-CCAGAAGAGCTGCGACATCC-3′ (100 nM), *CCR5R* 5′-GTTAAGGCTTTTACTCATCTCAGAAGCTAAC-3′ (100 nM), and *CCR5Pr* 5′-CalRed610-TTCCCCTACAAGAAACTCTCCCCGGTAAGTA-BHQ2-3′ (100 nM). All reactions were quantified on a QuantStudio 6 Pro Real-Time PCR System (Thermo Fisher Scientific) and expressed as cell equivalence per million cells.

### CMV immediate-early (IE) assay.

Cell-associated viral loads were determined using a qPCR targeting exon 1 of IE as previously described (81) with the following changes. Reactions were carried out using Fast Advance Mastermix (Applied Biosystems; catalog 4444964) in a 384-well plate. The reaction volume of 20 μL, 15 μL Mastermix plus 5 μL sample with a maximum of 0.25 μg being assayed per reaction. Samples were run in sextuplicate and analyzed by hybrid-digital analysis as previously described (82). Here we required signal from 2 out of 6 replicates for a sample to be considered positive. The limit of detection, based on assaying 0.25 μg DNA and the Poisson Distribution, is 1.6 copies IE per μg DNA.

### Proximity extension assay (Olink).

Plasma proteins from RMs were analyzed using a proximity extension assay (Olink Proteomics). Plasma was collected and stored as described above. The Olink Target 96 inflammation panel (Olink Proteomics) was used to measure protein levels following manufacturer’s instructions. In brief, pairs of oligonucleotide-labeled antibody probes are mixed with plasma to allow binding to their protein targets. The oligonucleotide pairs hybridize when the 2 compatible probes are in proximity. The reaction mixture, containing DNA polymerase, allows proximity-dependent DNA polymerization and the creation of a unique PCR target sequence; the amplified DNA sequence is quantified. Protein levels are expressed as arbitrary log_2_ transformed units, normalized protein expression (NPX). The following protein markers were included in the analysis: CXCL8, VEGF-A, GDNF, CDCP1, IL-7, OPG, LAP-TGF-β1, uPA, IL-6, CCL2, IL-17A, CXCL11, AXIN1, TNFSF10, IL-20RA, CXCL9, CST5, OSM, CXCL1, CCL4, CD6, SCF, IL-18, TGF-α, CCL13, CCL11, TNFSF14, FGF23, IL-10RA, FGF5, MMP-1, LIFR, FGF21, CCL19, IL-10RB, IL-18R1, PD-L1, TNFSF11, HGF, IL-12B, MMP-10, TNF-α, CCL23, CD5, CCL3, Flt3L, CXCL6, 4EBP1, SIRT2, CCL28, DNER, CD40, FGF19, CCL8, CASP8, CCL25, CX3CL1, TNFRSF9, NT-3, TNFSF12, CCL20, ST1A1, STAMBP, ADA, TNF-β, and CSF1.

### ELISAs.

IFN-γ plasma levels were measured using Rhesus Macaque IFN-gamma ELISA Kit (invitrogen; catalog EP8RB). The assay was conducted according to manufacturer’s instructions using undiluted samples.

IFN-α plasma levels were measured using Non-human IFN-alpha ELISA Kit (Novus Biologicals; catalog NBP3-11725). The assay was conducted according to manufacturer’s instructions using 1:2 diluted samples.

### Phenotyping characterization by flow cytometry.

Thawed, unstimulated rhesus macaque PBMCs, LN, BM, duodenum, and colon samples were stained to identify lymphocytes and monocytes. The samples were stained in predetermined optimal concentrations of the following antibodies: BB515 anti-Siglec-1 (CD169; BD Horizon Custom; clone 7-239; catalog 624279), BB630 anti-CD28 (BD Horizon Custom; clone CD28.2; catalog 624294), BB660 anti-CCR7 (CD197; BD Horizon Custom; clone 3D12; catalog 624295), BB700 anti-CD69 (BD OptiBuild; clone FN50; catalog 747520), BB790 anti-CXCR3 (CD183; BD Horizon Customs; clone 1C6/CXCR3; catalog 624296), BV421 anti-Granzyme B (BD Horizon; clone GB11; catalog 563389), BV510 LIVE/DEAD Fixable Aqua Dead Cell Stain (Invitrogen; catalog L34957), BV605 anti-CD14 (BioLegend; clone M5E2; catalog 301834), BV650 anti-PD-1 (CD279; BioLegend; clone EH12.2H7; catalog 329950), BV711 anti-HLA-DR (BD Horizon; clone G46-6; catalog 563696), BV750 anti-CD8a (BioLegend; clone SK1; catalog 344756), BV786 anti-CD16 (BD Horizon; clone 3G8; catalog 563690), BUV395 anti-CD3 (BD Horizon; clone SP34-2; catalog 564117), BUV496 anti-CD95 (BD Horizon Custom; clone DX2; catalog 624283), BUV563 anti-Bcl-2 (BD Horizon Custom; clone Bcl-2/100; catalog 624284), BUV615 anti-CCR6 (CD196; BD OptiBuild; clone 11A9; catalog 751515), BUV661 anti-CD25 (BD OptiBuild; clone 2A3; catalog 741685), BUV737 anti-CCR5 (CD195; BD OptiBuild; clone 3A9; catalog 748873), BUV805 anti-CD4 (BD OptiBuild; clone OKT4; catalog 750976), PE anti-FOXP3 (BioLegend; clone 206D; catalog 320108), PE-eFluor610 anti-CXCR5 (CD185; Invitrogen; clone MU5UBEE; catalog 61-9185-42), PE-Cy5 anti-CD127 (Invitrogen; clone eBioRDR5; catalog 15-1278-42), PE-Cy7 anti-α4β7 (NHPRR; clone A4B7R1; catalog PR-1427); APC anti-NKG2a (CD159a, Beckman Coulter; clone Z199; catalog A60797), R718 anti-Ki-67 (BD Horizon; clone B56; catalog 566963), APC-H7 anti-CD45 (BD Pharmingen Custom; clone D058-1283; catalog 624347). Foxp3/Transcription Factor Staining Buffer Set (eBioscience; catalog 00-5523-00) was used for permeabilization for Granzyme B, Bcl-2, FoxP3, and Ki-67. The samples were stained at 37°C for 15 min for the live/dead stain followed by 20 min for the surface antibodies and at 4°C for 30 min for the intracellular antibodies. The stained samples were fixed in 1% paraformaldehyde (PFA) and acquired on a Symphony A5 cytometer (BD Biosciences). Analysis was performed using FlowJo (version 10.10.0).

### Phenotypic analysis of human PBMCs.

Thawed, unstimulated human PBMCs were stained to identify CCR5 expression. The samples were stained in predetermined optimal concentrations of the following antibodies: BUV395 anti-CD4 (BD Horizon; clone L200; catalog 564107), BUV737 anti-CD3 (BD Horizon; clone UCHT 1; catalog 612750), BV605 anti-CD8 (BD Horizon; clone SK1; catalog 564116), BV650 anti-CD45RO (BD Horizon; clone UCHL1; catalog 563750), BV711 anti-CCR5 (BD Horizon; clone 2D7; catalog 563395), and BV510 LIVE/DEAD Fixable Aqua Dead Cell Stain (Invitrogen; catalog L34957). The samples were stained at 37°C for 15 min for the live/dead stain followed by 20 min for the antibodies. The stained samples were fixed in 1% PFA and acquired on a BD Fortessa cytometer (BD Biosciences). Analysis was performed using FlowJo (version 10.10.0).

### Activation-induced marker assay.

Thawed rhesus macaque PBMCs were stimulated with 3 μL CMV lysate (acquired by the ONPRC virology core) at 37°C for 16 hours. The samples were stained in predetermined optimal concentrations of the following antibodies: FITC anti-CD8a (BD Biosciences; clone SK1; catalog 347313), BB700 anti-CD69 (BD Horizon; clone FN50; catalog 747520), APC-H7 anti-CD45 (BD Pharmingen Custom; clone D058-1283; catalog 624347), BV421 anti-CD40L (CD154; BioLegend; clone 24-31; catalog 310824), BV510 LIVE/DEAD Fixable Aqua Dead Cell Stain (Invitrogen; catalog L34957), BV605 anti-CD28 (BD Horizon; clone CD28.2; catalog 562976), BV650 anti-CD4 (BD Horizon; clone L200; catalog 563737), PE-Cy7 anti-4-1BB (CD137; BioLegend; clone 4B4-1; catalog 309818), BUV395 anti-CD3 (BD Horizon; clone SP34-2; catalog 564117), BUV496 anti-CD95 (BD Horizon Custom; clone DX2; catalog 624283), and BUV737 anti-CCR5 (CD195; BD OptiBuild; clone 3A9; catalog 748873). The samples were stained at 37°C for 15 min for the live/dead stain followed by 20 min for the antibodies. The stained samples were fixed in 1% PFA and acquired on a Fortessa cytometer (BD Biosciences). Analysis was performed using FlowJo (version 10.10.0).

### Cell sorting for TCR sequencing.

Thawed rhesus macaque PBMCs were either immediately stained for the CCR5^+^CD4^+^ sorts or rested for 2 hours before adding anti-CD40 (Miltenyi Biotec; clone HB14; catalog 130-094-133) to a final concentration of 0.5 μg/mL followed by 3 μL RhCMV lysate (acquired by the ONPRC virology core) stimulation at 37°C for 16 hours for the CD4^+^ CMV-specific sort. Before staining CD4^+^ T cells were immuno-magnetically separated from bulk mononuclear cell suspensions by positive selection using the CD4^+^ T cell isolation kit, non-human primate, as per the manufacturer’s instructions (Miltenyi Biotec; catalog 130-092-144). The samples were stained in predetermined optimal concentrations of the following antibodies for the following 2 panels.

For CCR5^+^CD4^+^ T cell sorting: FITC anti-CD8a (BD Biosciences; clone SK1; catalog 347313), APC-H7 anti-CD45 (BD Pharmingen Custom; clone D058-1283; catalog 624347), BV510 LIVE/DEAD Fixable Aqua Dead Cell Stain (Invitrogen; catalog L34957), BV605 anti-CD28 (BD Horizon; clone CD28.2; catalog 562976), BV650 anti-CD4 (BD Horizon; clone L200; catalog 563737), BUV395 anti-CD3 (BD Horizon; clone SP34-2; catalog 564117), BUV496 anti-CD95 (BD Horizon Custom; clone DX2; catalog 624283), and BUV737 anti-CCR5 (CD195; BD OptiBuild; clone 3A9; catalog 748873).

For CD4^+^ CMV-specific T cell sorting: FITC anti-CD8a (BD Biosciences; clone SK1; catalog 347313), BB700 anti-CD69 (BD Horizon; clone FN50; catalog 747520), APC-H7 anti-CD45 (BD Pharmingen Custom; clone D058-1283; catalog 624347), BV421 anti-CD40L (CD154; BioLegend; clone 24-31; catalog 310824), BV510 LIVE/DEAD Fixable Aqua Dead Cell Stain (Invitrogen; catalog L34957), BV650 anti-CD4 (BD Horizon; clone L200; catalog 563737), PE-Cy7 anti-4-1BB (CD137; BioLegend; clone 4B4-1; catalog 309818), and BUV395 anti-CD3 (BD Horizon; clone SP34-2; catalog 564117).

The samples were stained at 37°C for 15 min for the live/dead stain followed by 20 min for the antibodies. The stained samples were prepared in RPMI 1640 medium (no phenol red; Gibco; catalog 11835030) with 10% FBS at a final concentration of 10 million cells/mL and then sorted on a FACSAria Fusion (BD Biosciences). Sorted samples were collected in RA1 lysis buffer (Takara Bio; catalog 740961) in a final volume of 350 μL.

### RNA isolation and TCR sequencing.

Collected samples were homogenized with a 20-gauge needle and syringe. RNA isolation on homogenized samples was performed using the RNeasy Micro Kit (QIAGEN; catalog 74004), with the addition of 350 μL RLT and 10% 2-mercaptoethanol (MilliporeSigma; catalog M6250), following manufacturer’s instructions. TCR library generation was performed using SMARTer Human TCR a/b profiling kit v2 (TCRv2; Takara Bio; catalog 634478). Generated indexed libraries were sequenced on a MiSeq platform (Illumina) using a v3 reagent kit (600 cycles; Illumina; catalog MS-102-3003) with 2 × 300 bp paired-end reads.

TCR-sequencing data were processed using Cogent NGS Immune Profiler Software (version 2.0) with standard parameters and the human genome as the alignment reference, in accordance with the TCRv2 protocol. To ensure comparability across samples, TCR datasets were downsampled to the same number of clones per sample prior to repertoire analysis.

### Statistics.

R (version 4.5.0) (83) was used to perform statistical analysis and graph creation. The packages ggpubr (version 0.6.0), ggplot2 (version 3.5.0) (84), ComplexHeatmap (version 2.18.0) (85), and circlize (version 0.4.16) (86) were used for graph creation, and immunarch (version 0.9.1) was used for TCR repertoire analysis. Protein-protein interaction analysis was performed using STRING (Search Tool for the Retrieval of Interacting Genes/Proteins; version 12.0) (87). Wilcoxon matched pairs signed-rank test was used for non-parametric paired analysis, paired *t* test was used for parametric paired analysis, Mann-Whitney *U* test for non-parametric unpaired analysis, Spearman’s rank correlation for non-parametric correlation, and Shapiro-Wilk test for normality. Differences in categorical variables were analyzed using the χ^2^ test. Statistical significance is indicated by *P* < 0.05. All statistical tests are 2 sided.

### Study approval.

The study underwent review and approval by the institutional Animal Care and Use Committee of Tulane University. Animal care procedures adhered to the guidelines outlined in the NIH’s *Guide for the Care and Use of Laboratory Animals* (National Academies Press, 2011). Handling procedures and containment protocols for animals were approved by the Tulane University Institutional Biosafety Committee, ensuring compliance with Biosafety Level 2 containment standards. Furthermore, the Tulane National Primate Research Center holds full accreditation from the Association for Assessment and Accreditation of Laboratory Animal Care, underscoring its commitment to maintaining high standards of animal welfare and research ethics. Human studies were approved by the Institutional Review Board of University Hospitals Cleveland Medical Center, Cleveland, Ohio, USA (IRB # 01-98-55).

### Data availability.

The authors declare that data supporting the results and conclusions of this study are available within the paper and its supplement. The Supporting Data Values file contains all data points shown in graphs. Additional data that support the findings of this study are available from the corresponding authors upon reasonable request. The TCR-sequencing data supporting this study have been deposited in the NCBI Gene Expression Omnibus under accession number GSE297607. Codes used for data analysis are found here: https://github.com/chrysperdios/RhCMV-Expands-CCR5-Memory-T-Cells-and-promotes-SIV-reservoir-seeding-in-the-Gut-Mucosa, commit ID ae8dfc4.

## Author contributions

JCM designed the animal study. CP and JCM designed the laboratory study. CP, CDC, NSB, ATB, and SNA participated in tissue acquisition and processing. CMF and BFK provided SIVmac239M for study infection. CP, CDC, JCM, and NSB performed experiments. MJM, CA, and LAR provided technical expertise. CP and JCM analyzed and interpreted data. AK provided helpful discussion and assisted with data interpretation. LDM provided veterinary support. CP and JCM wrote the manuscript. MLF acquired and analyzed human samples. JCM, CDC, MLF, NSB, and ATB provided critical and substantive intellectual editing. CP and JCM prepared manuscript figures. All authors approved the manuscript.

## Funding support

This work is the result of NIH funding, in whole or in part, and is subject to the NIH Public Access Policy. Through acceptance of this federal funding, the NIH has been given a right to make the work publicly available in PubMed Central.

- NIH base grant to the TNPRC P51 OD011104.
- NIH grants R01 AI167644 and R21 OD031229 (to JCM).
- NIH grant S10 OD026800 to support the TNPRC Flow Cytometry Core.
- NIH base grant OD011104 to support the TNPRC Pathogen Detection and Quantification Core.
- National Cancer Institute, NIH, under Contract No. 75N91019D00024.
- NIH grant P51 OD011092 to ONPRC Viral Immunology Core.

## Supplementary Material

Supplemental data

Supporting data values

## Figures and Tables

**Table 1:**
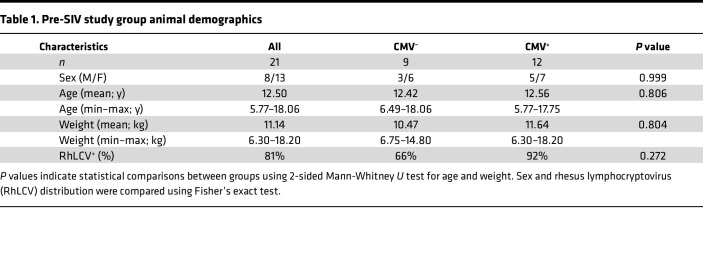
Pre-SIV study group animal demographics

## Version 1 11/25/2025

In-Press Preview

## Version 2 01/09/2026

Electronic publication
