# Supplementary material for: RhCMV expands CCR5+ memory T cells and promotes SIV reservoir seeding in the gut mucosa
Source: JCI Insight. 2025 Nov 25;11(1):e198743. doi: 10.1172/jci.insight.198743 (PMC12890476; doi:10.1172/jci.insight.198743)
Supplement: Supplemental data [file jciinsight-11-198743-s085.pdf]

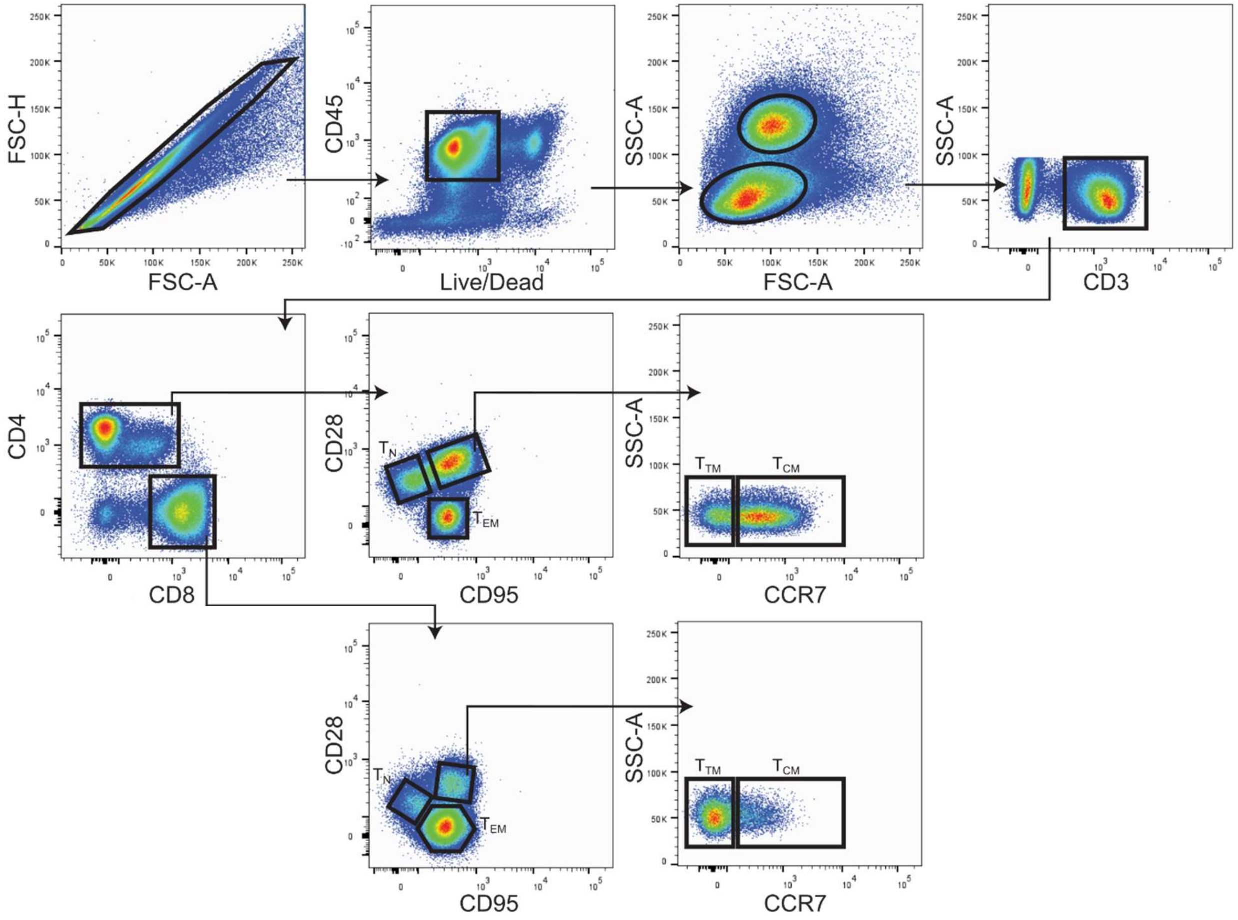

Figure S1. Gating strategy for $CD4^+$ and $CD8^+$ T cell maturation subsets. Representative flow cytometry plots illustrating the gating strategy used to define $CD4^+$ and $CD8^+$ T cell subpopulations based on maturation state. Key: T_N, naive; T_{CM}, central memory; T_{TM}, transitional memory; T_{EM}, effector memory.

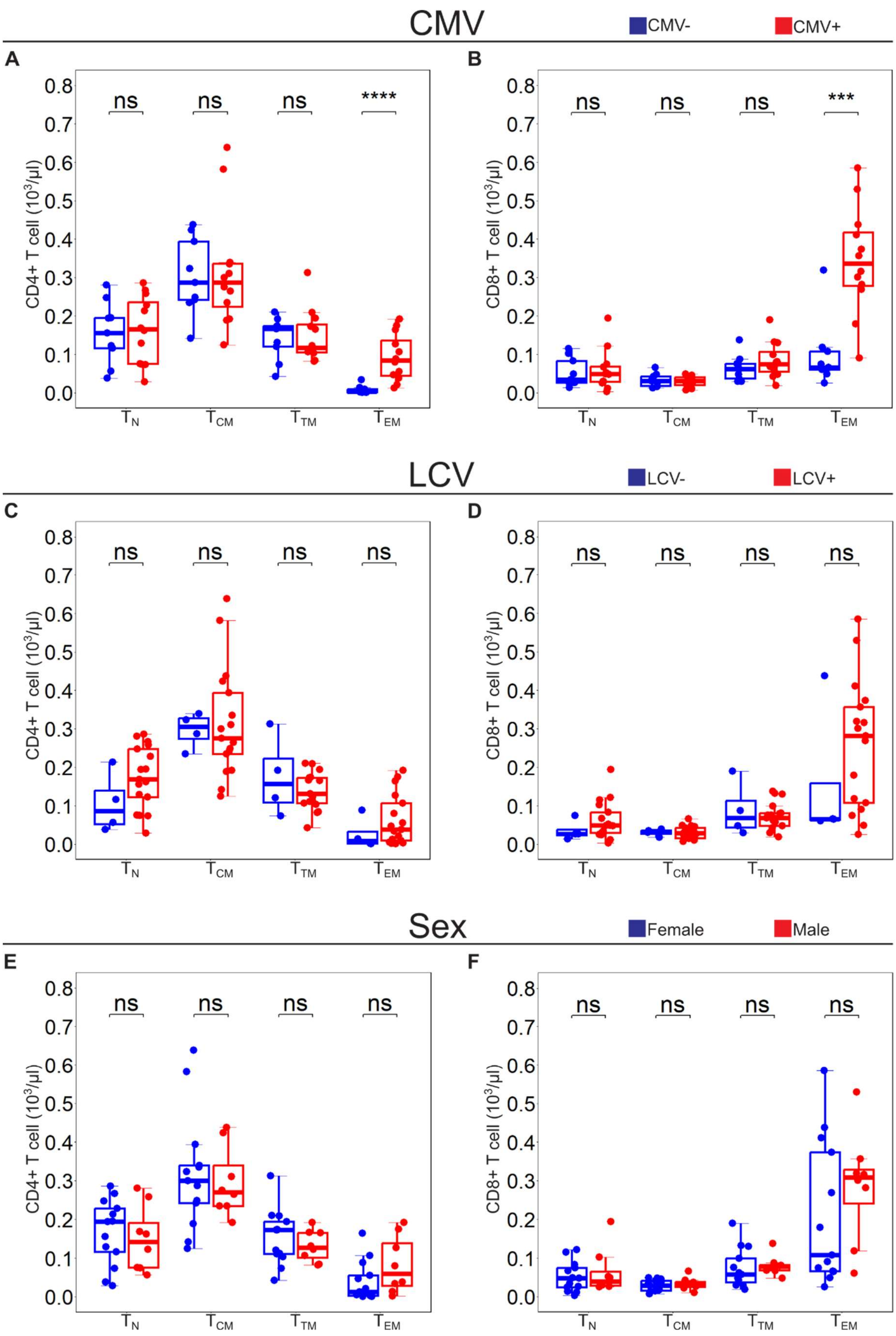
CMV
CMV-
CMV+
A
B
ns
****
***
CD4+ T cell (10^3/µl)
CD8+ T cell (10^3/µl)
LCV
LCV-
LCV+
C
D
Sex
Female
Male
E
F

Figure S2. Circulating CD4$^+$ and CD8$^+$ T cell maturation subset distributions by RhCMV- and RhLCV- serostatus, and sex pre-SIV. Blood CD4+ (**A**) and CD8+ (**B**) T cell numbers across different maturation subpopulations by RhCMV-serostatus (RhCMV+ n = 12, RhCMV- n = 9). Blood CD4+ (**C**) and CD8+ (**D**) T cell numbers across different maturation subpopulations by RhLCV-serostatus (RhLCV+ n = 17, RhLCV- n = 4). Blood CD4+ (**E**) and CD8+ (**F**) T cell numbers across different maturation subpopulations by sex (Female n = 13, Male n = 8). Error bars represent 1.5 times the interquartile range. Statistical comparison performed using two-sided Mann-Whitney U test. Key: T_N, naive; T_{CM}, central memory; T_{TM}, transitional memory; T_{EM}, effector memory; ns $p>0.05$; *** $p<0.001$; **** $p<0.0001$.

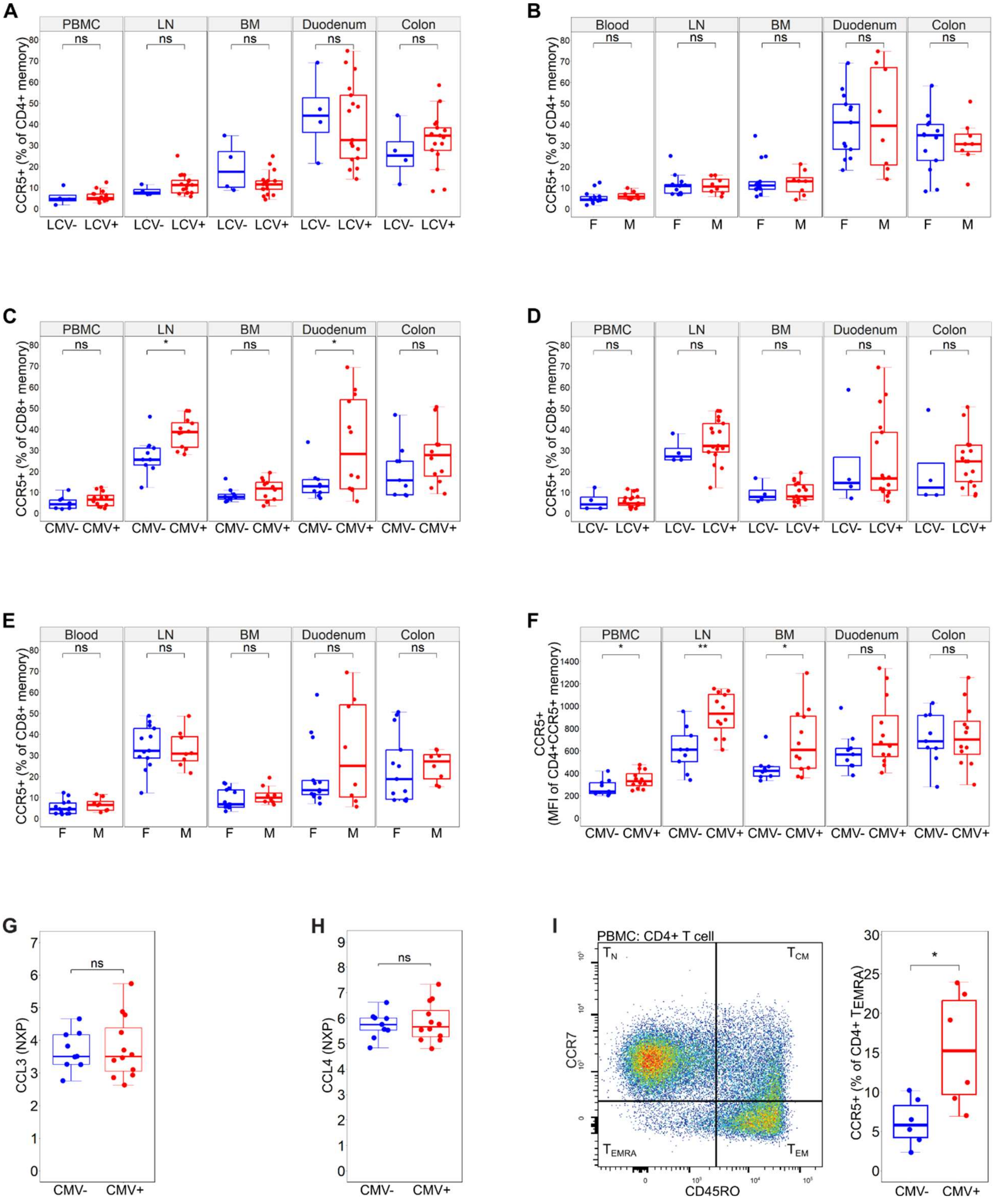

Figure S3. CCR5 expression on memory T cells and plasma chemokine levels by viral serostatus and sex. Pre-SIV %CCR5+ CD4+ memory cells by **A** RhLCV-serostatus and **B** sex.

Pre-SIV %CCR5+ CD8+ memory cells by **C** RhCMV-serostatus, **D** RhLCV-serostatus and **E** sex. **F** Pre-SIV CCR5+ MFI of CD4+CCR5+ memory cells by RhCMV-serostatus**.** **G** Pre-SIV plasma CCL3 expression by RhCMV-serostatus. **H** Pre-SIV plasma CCL4 expression by RhCMV-serostatus. **I** HIV-uninfected human PBMC CD4+ T cell maturation subpopulation gating strategy and %CCR5 CD4+ T_{EMRA} cells. For all figures RhLCV+ n = 17, RhLCV- n = 4; RhCMV+ n = 12, RhCMV- n = 9; F = 13 M = 8. Error bars represent 1.5 times the interquartile range. Statistical comparison performed using two-sided Mann-Whitney U test. Key: PBMC, peripheral blood mononuclear cells; LN, lymph node; BM, bone marrow; MFI, median fluorescence intensity; NPX, normalized protein expression; F, female; M, male; T_N, naive; T_{CM}, central memory; T_{EM}, effector memory T_{EMRA}, terminal effector memory; ns p>0.05; * p<0.05; ** p<0.01.

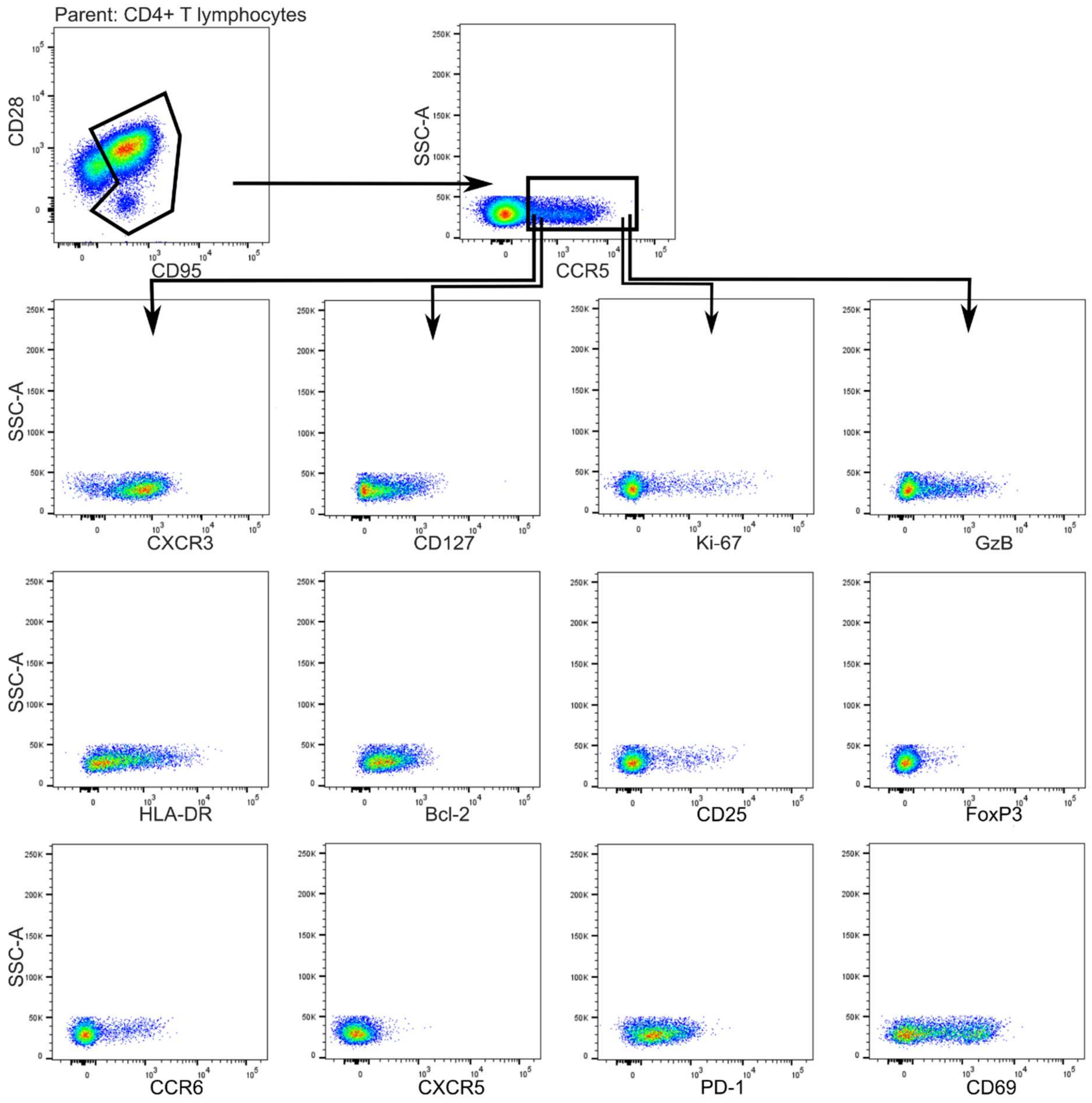

Figure S4. Gating strategy for CCR5^{+}CD4^{+} memory T lymphocytes. Representative flow cytometry plots showing sequential gating. CD4^{+} T cells were first gated for the memory subset using CD28 and CD95 expression. Within this population, CCR5^{+} cells were identified and further characterized for expression of markers of interest. This strategy defines functional and phenotypic features of CCR5^{+}CD4^{+} memory T cells.

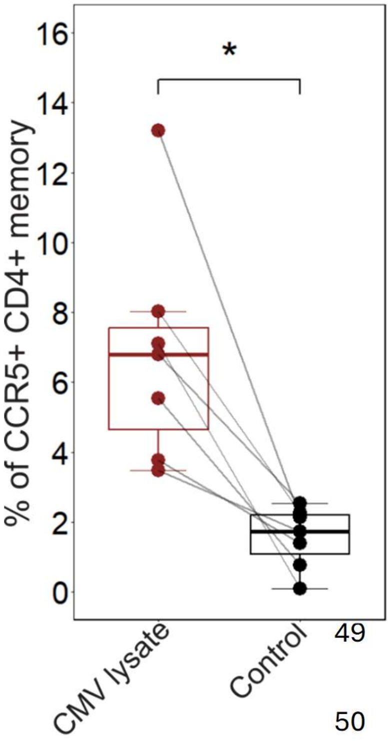

Figure S5. CMV lysate vs control stimulation of CCR5⁺CD4⁺ memory T cells. Quantification of CD40L⁺CD69⁺CCR5⁺CD4⁺ memory PBMCs from RhCMV+ animals cultured with and without CMV lysate (n=7). Error bars represent 1.5 times the interquartile range. Statistical comparison performed using two-sided Mann-Whitney U test.

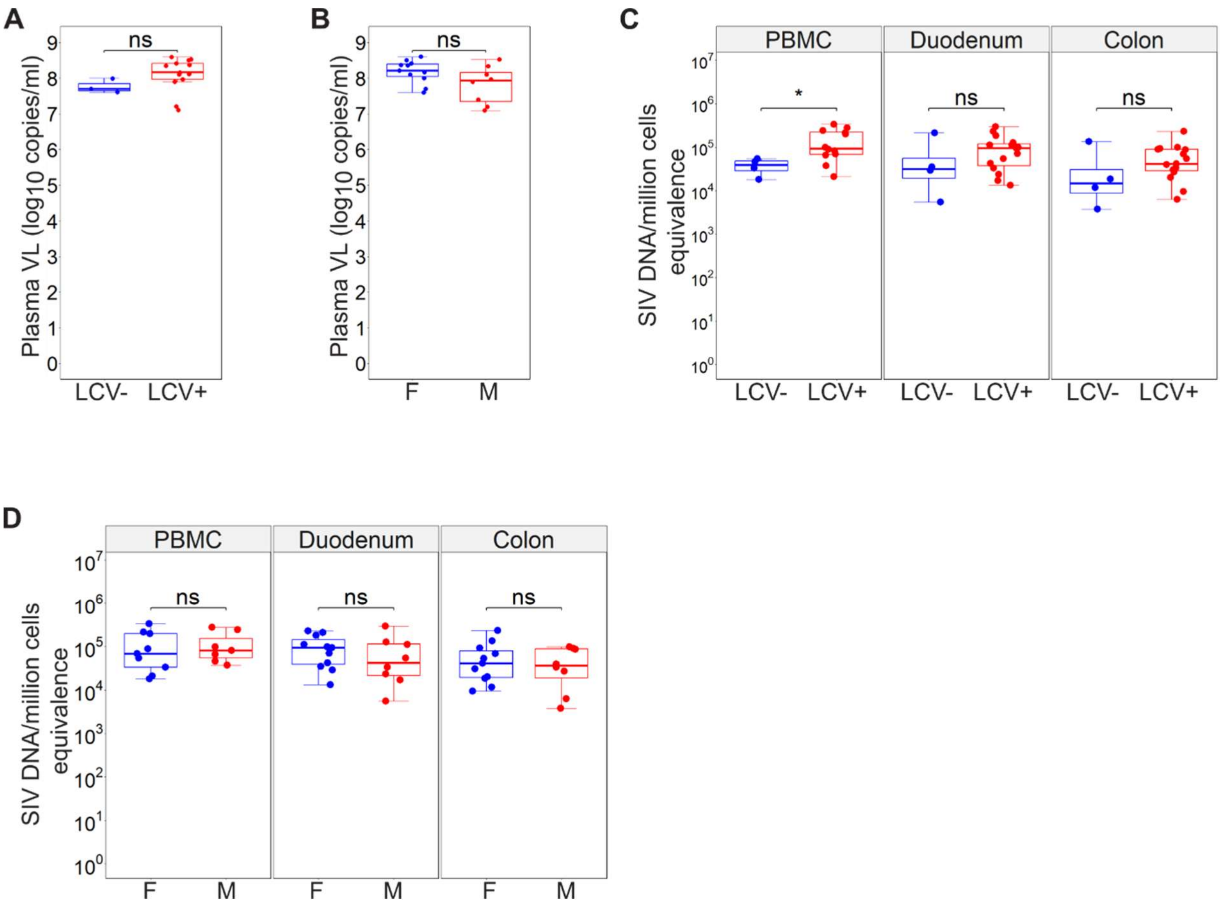

Figure S6. Plasma and cell-associated SIV levels by RhLCV serostatus. Plasma viral loads (VL) on day 13/14 post-SIV by **A** RhLCV-serostatus and **B** sex. Cell-associated SIV DNA metrics on day 13/14 post-SIV by **C** RhLCV-serostatus and **D** sex. For all graphs RhLCV+ n = 15; RhLCV- n = 4; F = 11; M = 8. Error bars represent 1.5 times the interquartile range. Statistical comparison performed using two-sided Mann-Whitney U test. Key: PBMC, peripheral blood mononuclear cells; F, female; M, male; ns $p>0.05$; * $p<0.05$.

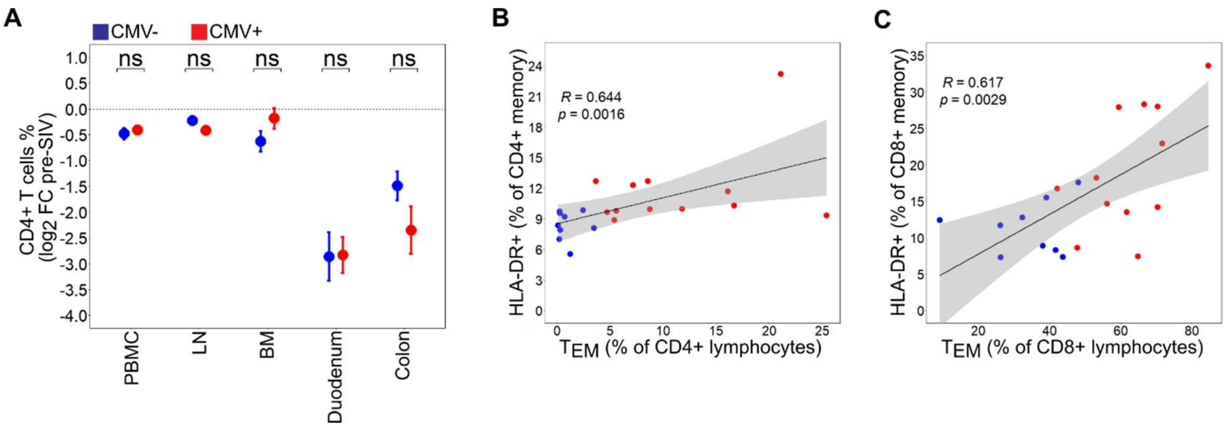

Figure S7. CD4+ T cell abundance and associations between activation and effector memory. A Log_2 ratios of %CD4+ T cells, mean ± standard error, post-SIV infection relative to pre-SIV baseline across sampled tissues by RhCMV-serostatus (RhCMV+ n = 11; RhCMV- n = 8). **B** Two-sided Spearman's correlation between pre-SIV %HLA-DR+ CD4+ memory T cells against %T_{EM} CD4+ T lymphocytes (RhCMV+ n = 12; RhCMV- n = 9). **C** Two-sided Spearman's correlation between pre-SIV %HLA-DR+ CD8+ memory T cells against %T_{EM} CD8+ T lymphocytes (RhCMV+ n = 12; RhCMV- n = 9). For A statistical comparison performed using two-sided Mann-Whitney U test. For B-C shaded area represents 95% confidence interval. Key: PBMC, peripheral blood mononuclear cells; LN, lymph node; BM, bone marrow; T_{EM}, effector memory; ns p>0.05.

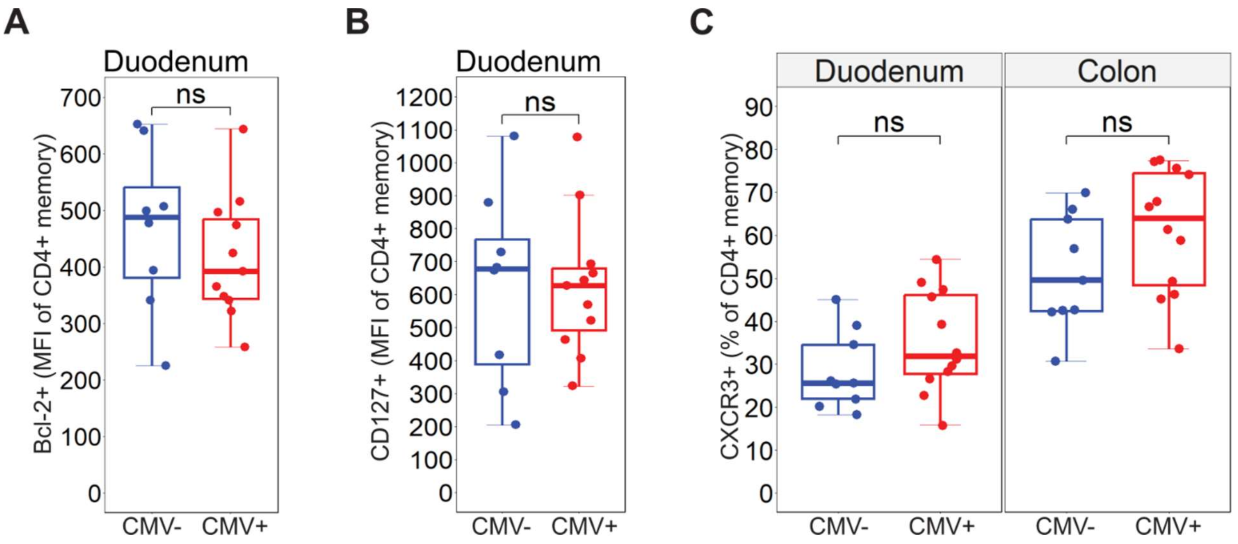

Figure S8. Markers of survival and trafficking in gut CD4+ memory T cells. A Bcl-2+ CD4+ memory T cell median fluorescent intensity (MFI) by RhCMV-serostatus. **B** CD127+ CD4+ memory T cell MFI by RhCMV-serostatus. **C** %CXCR3+ CD4+ memory T cell across duodenum and colon by RhCMV-serostatus. For all graphs RhCMV+ n = 11; RhCMV- n = 8. For A, B data was taken during 13/14 days post SIV infection. For C, data was taken at pre-infection. Error bars represent 1.5 times the interquartile range. Statistical comparison performed using two-sided Mann-Whitney U test. Key: NPX, normalized protein expression; ns p>0.05.

A

CD8+ T_{EM}% Quartile Demographics

Characteristics	All	Q1	Q2	Q3	Q4	p-value
n	46	12	11	11	12	
Age (mean; years)	12.22	13.17	11.74	11.53	12.33	0.3013
Age (min-max; years)	8.28-17.43	8.40-15.63	8.28-17.43	9.28-15.28	8.55-15.38	
CD8+ T_{EM} % (mean)	46.63	29.45	42.13	51.93	63.1	<0.0001
CD8+ T_{EM} % (min-max)	20.3-74.1	20.3-36.1	38.6-47.2	48.2-54.9	55.4-74.1	
Sex(M/F)	29/17	6/6	9/2	5/6	9/3	0.192

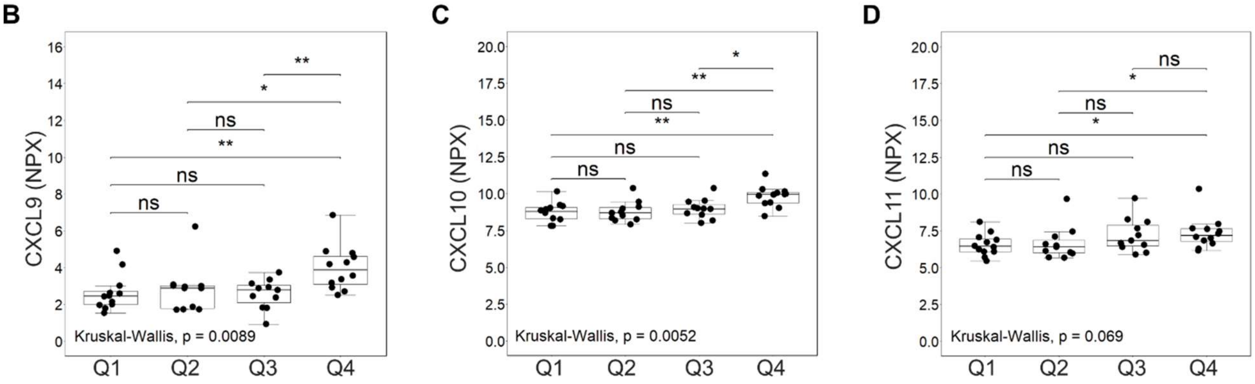

Figure S9. CXCL9/10/11 expression stratified by CD8+ TEM frequency in SIV-RhCMV+ rhesus macaques. A Age and sex characteristics of RhCMV+ rhesus macaques from the large animal cohort divided by % T_{EM}CD8+ T cells quartiles. **B-D** Expression of CXCL9/10/11 by %T_{EM} CD8+ T cells quartiles. Sex distribution was compared using the chi-square test. Statistical comparison performed using Kruskal-Wallis test and post-hoc analysis by two-sided Dunn's test. Error bars represent 1.5 times the interquartile range. Key: T_{EM}, effector memory; NPX, normalized protein expression; ns p> 0.05; * p<0.05 ** p<0.001.
